# Causal relationship between albumin, total protein, and colorectal cancer risk: A 2-sample Mendelian randomization study

**DOI:** 10.1097/MD.0000000000045229

**Published:** 2025-10-17

**Authors:** Jia Chen, Xin Zhong, Fulin Wang, Yong Kuang, Jiong Chen

**Affiliations:** a Digestive Diseases Center, The Seventh Affiliated Hospital, Sun Yat-sen University, Shenzhen, Guangdong, People’s Republic of China; b Guangdong Provincial Key Laboratory of Digestive Cancer Research, The Seventh Affiliated Hospital of Sun Yat-sen University, Shenzhen, Guangdong, People’s Republic of China; c Department of Anaesthesiology, The Seventh Affiliated Hospital of Sun Yat-sen University, Shenzhen, Guangdong, People’s Republic of China.

**Keywords:** albumin, colorectal cancer, genome-wide association studies, Mendelian randomization, total protein

## Abstract

Albumin (ALB) and total protein (TP) are vital constituents of the blood, and their levels and roles in the risk of colorectal cancer (CRC) are of significance. Previous observational studies have reported correlations among ALB, TP, and CRC. However, the existence of a causal relationship between ALB and CRC in European populations has not been adequately investigated and the causal link between TP and CRC remains unexplored. To address these gaps, we applied Mendelian randomization (MR) to investigate the potential causal relationship between ALB, TP, and CRC. Two-sample MR analysis was used to investigate whether there was a causal relationship between ALB, TP, and CRC. Our exposure data were extracted from genome-wide association study (GWAS) databases sourced from the UK Biobank, containing 315,268 and 314,921 Europeans participants for ALB and TP analyses, respectively. Single nucleotide polymorphisms that were significantly associated with ALB and TP were assessed using GWAS datasets. Our data were derived from the FinnGen Consortium CRC GWAS, which contained 6509 CRC cases and 28,7137 controls. Causal inference between ALB, TP, and CRC was performed using 3 MR methods: inverse variance weighting (IVW), MR-Egger, and weighted median. The IVW analysis showed no significant causal association between ALB and CRC (OR = 1.04, 95% CI = 0.89–1.21, *P* = .65). In contrast, the IVW analysis for TP and CRC showed a significant causal association (OR = 0.78, 95% CI = 0.66–0.92, *P* = .003), suggesting a reduced risk of CRC. Through a 2-sample MR study investigating the causal relationship between ALB, TP, and CRC in a European population, our findings revealed a significant causal relationship between TP and a reduced risk of CRC.

## 1. Introduction

Colorectal cancer (CRC) is currently the 3rd most common neoplasm and the 2nd leading cause of cancer-related deaths worldwide, as reported by Global Epidemiology.^[1]^ In 2020, there were more than 1.9 million new cases and 0.9 million deaths from CRC.^[2]^ By 2030, new cases and deaths from CRC are projected to exceed 2.2 million and 1.1 million,^[3]^ respectively. CRC has a slow onset and good early prognosis. Enhancing early screening for CRC can effectively reduce the burden of the disease and improve the survival rate of CRC patients.^[4,5]^ Colorectoscopy, the gold standard for diagnosing CRC, reduces population compliance owing to its invasive nature and complex bowel preparation. Therefore, we attempted to find more convenient supplementary screening methods for CRC.

Albumin (ALB) is the body’s predominant serum protein,^[6]^ accounting for approximately 50% of total plasma protein.^[7]^ It is mainly synthesized by the liver and is involved in a wide range of physiological processes, including maintenance of colloid osmolality, binding function, and antioxidant activity.^[8]^ ALB and total protein (TP) are commonly used clinical assays to reflect nutritional status, inflammation, and liver function.^[9,10]^ There are a large number of clinical studies examining the relationship between ALB and CRC.^[11,12]^ There is a lack of uniformity in the studies on the relationship between ALB and CRC. One study found no significant correlation between ALB and CRC.^[13]^ Other studies proved that ALB showed a negative correlation with CRC.^[11,14]^ One study showed a positive correlation between ALB and left-sided distal CRC.^[15]^ Regarding the causal relationship between ALB and CRC, a previous study on East Asians confirmed a negative association between ALB and CRC.^[9]^ To the best of our knowledge, there have been no studies on the causal relationship between ALB and CRC in Europeans. Regarding the relationship between TP and CRC, previous studies have demonstrated a significant negative correlation between TP and CRC.^[14]^ To our knowledge, a causal relationship between TP and CRC has not been reported.

Mendelian randomization (MR) is a method for exploring causal relationships between exposure and outcome.^[16]^ MR uses the random assignment of single nucleotide polymorphisms (SNPs) from parent to offspring and can minimize the effects of confounders and reverse causation.^[17]^

In this study, we explored the potential causal relationship between ALB, TP, and CRC, using a 2-sample MR approach.

## 2. Materials and methods

### 2.1. Research design

**Figure 1.**
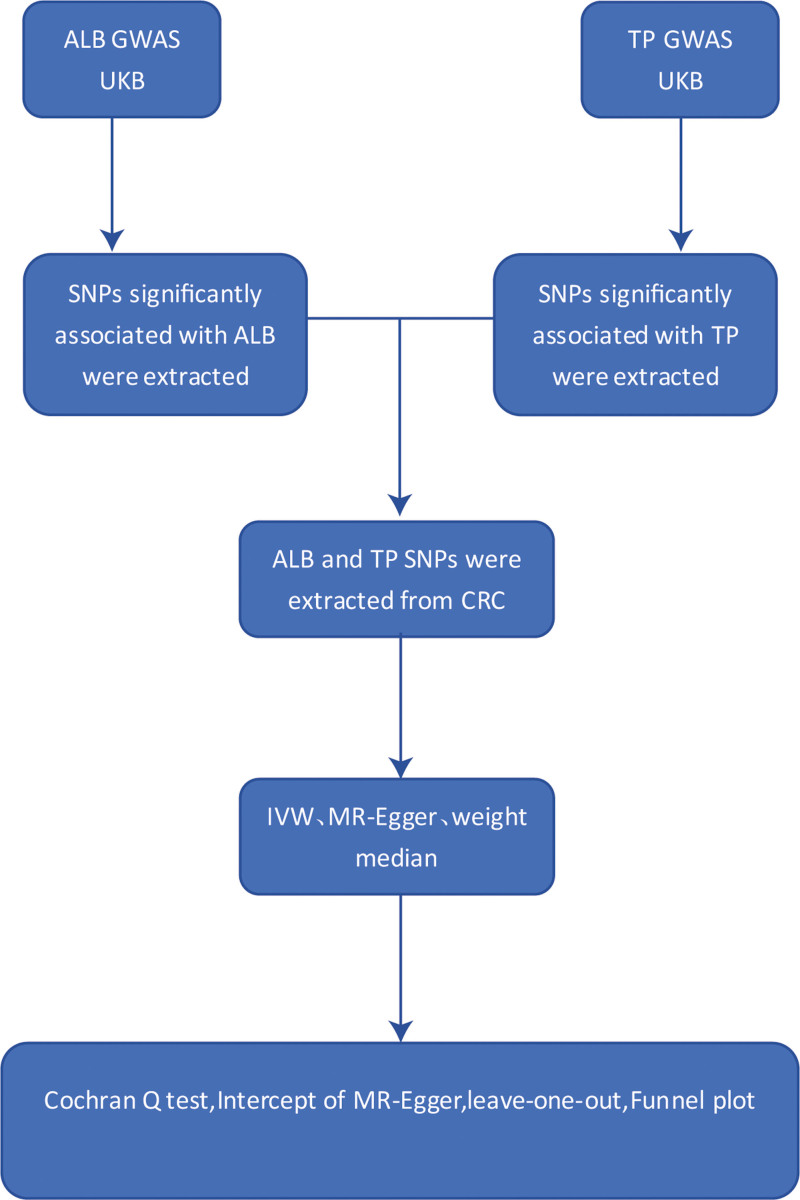
Overview of the Mendelian randomization (MR) study design for ALB and TP on CRC. ALB = albumin, CRC = colorectal cancer, TP = total protein.

**Figure 2.**
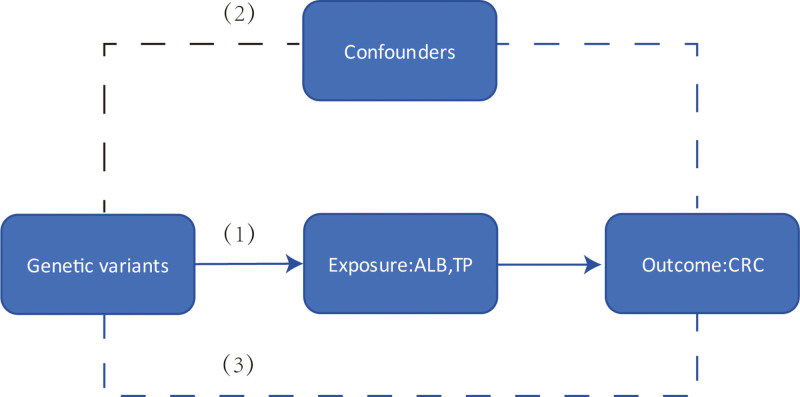
Three core assumptions of the Mendelian randomization (MR) framework.

The flow of this study is summarized in Figure 1. In this study, we used a 2-sample MR approach, examining the genome-wide association study (GWAS) dataset from the UK BioBank as the “exposure” and GWAS dataset from the FinnGen Consortium as the ‘outcome,’ to explore the causal relationship between ALB and TP and CRC. This study is based on 3 core assumptions of MR (Fig. 2): hypothesis of correlation: the selected genetic variants are significantly correlated with the exposure of interest; independence hypothesis: the selected genetic variants were not correlated with any confounders of exposure or outcome; exclusivity hypothesis: genetic variants act on the outcome only through exposure.^[18,19]^

### 2.2. Data sources

Genetic variation in ALB was obtained from the GWAS dataset published in the UK Biobank, which contained 315,268 participants of European ethnicity, providing a total of 135,853,334 SNPs. Genetic variation in TP was also derived from a GWAS dataset published in the UK Biobank, which contained 314,921 participants of European ethnicity and provided a total of 13,585,298 SNPs. Pooled data for CRC were obtained from the FinnGen Consortium using the latest version of the FinnGen Consortium (version R9), which contains genotype data from the Finnish Biobank as well as data from the Finnish Information Registry. It can be used to study the relationship between genetic variations and disease patterns. In this time, 6509 CRC cases and 287,137 control cases were identified. The ethics of the CRC GWAS was approved by the FinnGen Steering Committee, and each participant signed an informed consent form.

### 2.3. Instrumental variable selection

To investigate the causal relationship between ALB, TP, and CRC, SNPs highly associated with ALB, TP, and CRC were selected using *P* < 5 × 10^-8^ a genome-wide significance threshold. LD clumping was performed using a threshold of *r*² < 0.001 and a window size of 10,000 kb to ensure the independence of selected SNPs. To avoid bias caused by weakly correlated instrumental variables, the *F*-value was calculated for each SNP, and *F*-values >10 were considered strongly correlated. Table S1A, Supplemental Digital Content, https://links.lww.com/MD/Q363 shows SNP information for ALB and CRC. Table S1B, Supplemental Digital Content, https://links.lww.com/MD/Q363 shows SNP information for TP and CRC. The *F*-values for all selected SNPs are provided in Table S2A (for ALB) and Table S2B (for TP), Supplemental Digital Content, https://links.lww.com/MD/Q363.

Although this study involves 2 exposures (ALB and TP), we believe that no additional Bonferroni correction is necessary. Firstly, the genome-wide significance threshold (*P* < 5 × 10⁻⁸) already accounts for strict multiple testing correction across millions of SNPs, which is more conservative than applying a Bonferroni correction for 2 exposures (*P* < .025). Secondly, the genetic instruments for ALB and TP were derived through completely independent selection processes, with no overlap in genomic locations or functions between the 2 sets of SNPs (all pairwise *r*² < 0.001), making their statistical tests independent of each other. Finally, Bonferroni correction assumes all tests are fully correlated, but in the case of independent instrumental variables, excessive correction could lead to a reduction in statistical power.

### 2.4. Two-sample MR analysis

Our 2-sample MR study was conducted using the R software (version 4.2.1; R Foundation for Statistical Computing, Vienna, Austria) and the TwoSampleMR package. After extracting SNPs significantly associated with exposure and outcome, 3 MR methods (IVW, MR-Egger, and weighted median) were used to investigate the causal relationship between ALB, TP, and CRC to eliminate the effects of horizontal pleiotropy and heterogeneity. We mainly applied IVW to our analyses. IVW uses a meta-analysis approach to pool the Wald ratios corresponding to each SNP and assess consistency in the causal relationship between exposure and outcome.^[20]^ IVW’s random effects model allows SNPs with horizontal pleiotropy to be included in the analysis, returning unbiased estimates when the horizontal pleiotropy of all SNPs is balanced.^[21]^ Although IVW’s inclusion of more SNPs in IVW effectively improves statistical efficacy, it may lead to the inclusion of invalid instrumental variables.^[22]^ The MR-Egger weighted median was used as a complement to the IVW. The MR-Egger weighted median allows for more reliable assessments over a wider range of scales, but it has lower statistical efficacy than IVW.^[23]^ MR-Egger test allows genetic variation to have multiple validity, provided that the effect does not affect the relationship between genetic variation and exposure.^[24]^ The weighted median provides an assessment of concordance when no <50% of the genetic variation is valid.^[25]^ MR-Egger test was used to test for the presence of horizontal pleiotropy if *P* < .05, indicating the presence of horizontal pleiotropy. The leave-one-out method of the IVW was used to test the stability of causality.

## 3. Results

### 3.1. There was no significant pleiotropy among the ALB and TP SNPs selected from the CRC GWAS dataset

The results of the MR-Egger _intercept test showed that the *P*-values for ALB and TP were .12 and .94, respectively, and the *P*-values were all >.05, so there was no significant pleiotropy among the ALB and TP SNPs selected from the CRC GWAS dataset. We considered SNPs to be independent of confounders, consistent with the second hypothesis of MR. Therefore, all ALB and TP SNPs were included in this TwoSample MR analysis.

### 3.2. There was significant heterogeneity among ALB and TP SNPs in CRC GWAS dataset

The Cochran Q test results for both MR-Egger and inverse variance weighted (IVW) methods yielded *P*-values below .05, indicating significant heterogeneity among the instrumental variables for both ALB and TP. Specifically, for ALB, Cochran Q was 225.118 (df = 155, *P* = 2.00 × 10⁻⁴) with MR-Egger and 228.716 (df = 156, *P* = 1.33 × 10⁻⁴) with IVW. For TP, the values were 306.318 (df = 174, *P* = 2.36 × 10⁻^9^) for MR-Egger and 306.327 (df = 175, *P* = 3.15 × 10⁻^9^) for IVW. Given the presence of substantial heterogeneity in the CRC GWAS dataset for SNPs associated with ALB and TP, the random effects IVW model was primarily employed for the MR analysis.

### 3.3. The results were robust

**Figure 3.**
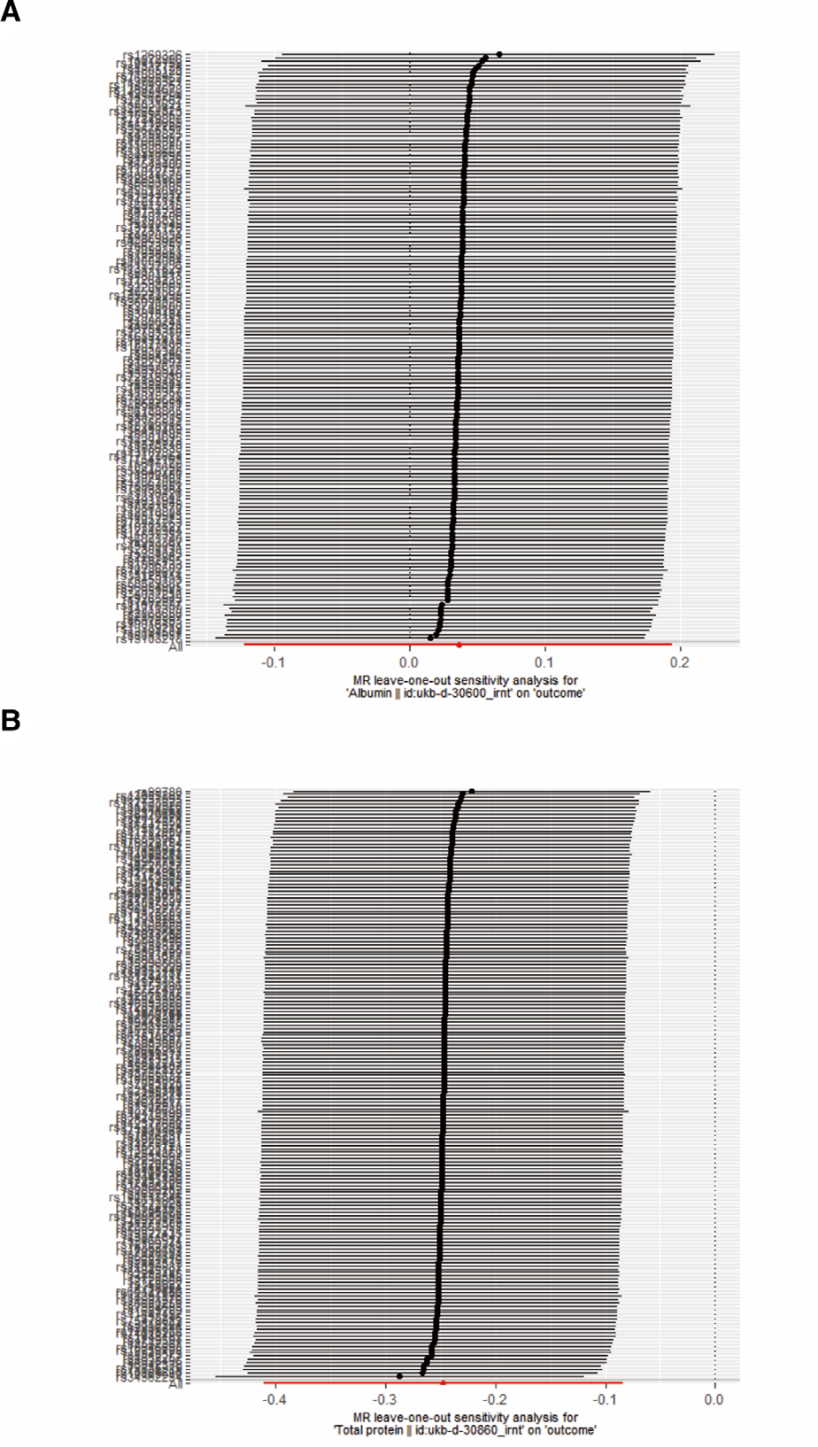
Leave-one-out detection plot of (A) ALB and (B) TP versus CRC. ALB = albumin, CRC = colorectal cancer, TP = total protein.

**Figure 4.**
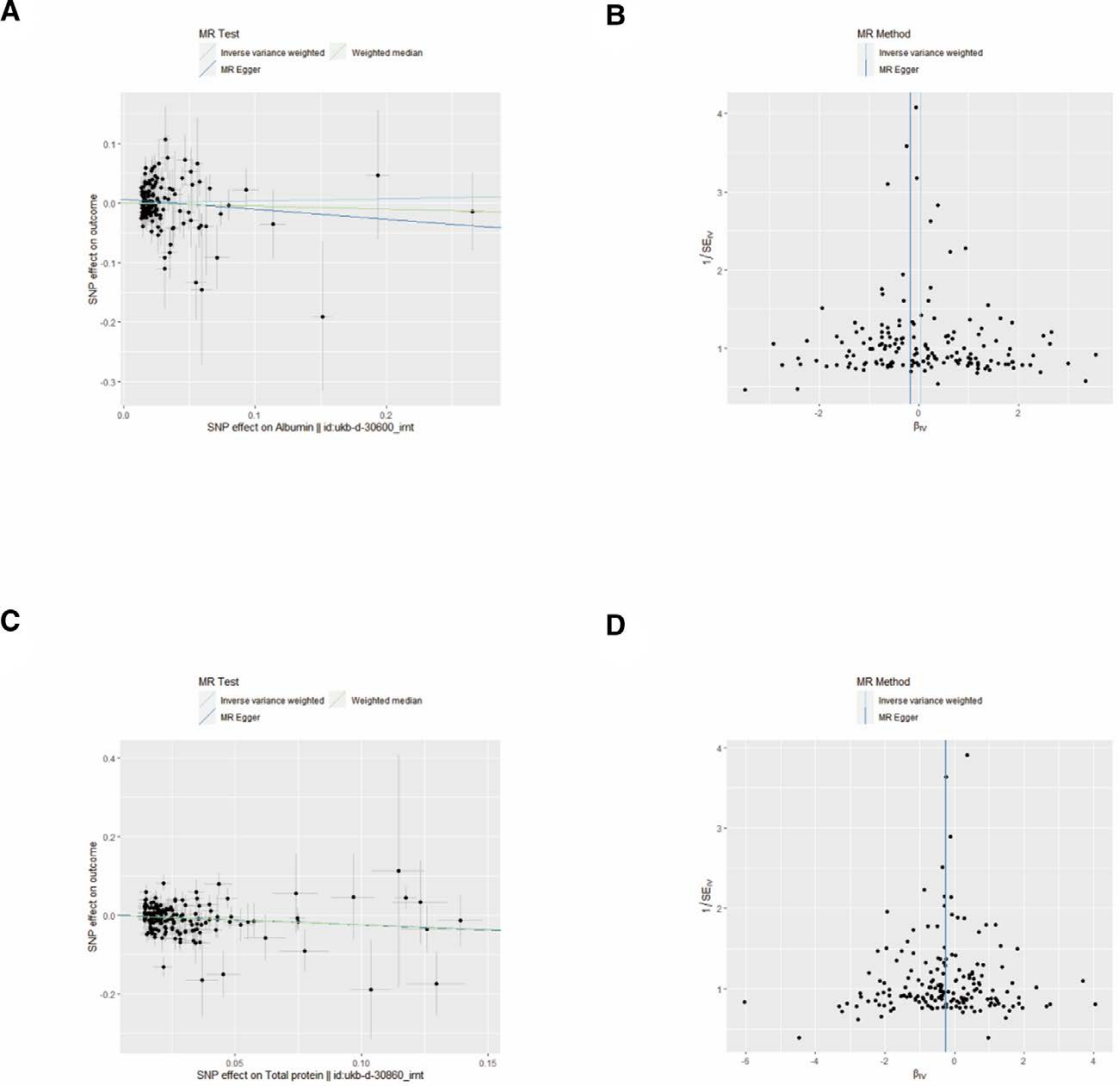
Scatter plots and funnel plots from the Mendelian randomization analysis assessing the causal effects of ALB and TP on CRC. (A) Scatter plot showing the causal effect of genetically predicted ALB on CRC risk. (B) Funnel plot for the ALB-associated genetic instruments. (C) Scatter plot showing the causal effect of genetically predicted TP on CRC risk. (D) Funnel plot for the TP-associated genetic instruments. ALB = albumin, CRC = colorectal cancer, TP = total protein.

Figure 3 shows the leave-one-out susceptibility analysis of ALB and TP SNPs in CRC. This shows that no SNPs can significantly change the causal relationship between TP and CRC, indicating that the results are robust. Egger intercepts did not indicate significant pleiotropy, and the funnel plot was symmetrical on both sides (Fig. 4).

### 3.4. MR analysis

#### 3.4.1. There was no significant causal association between ALB and CRC

**Figure 5.**
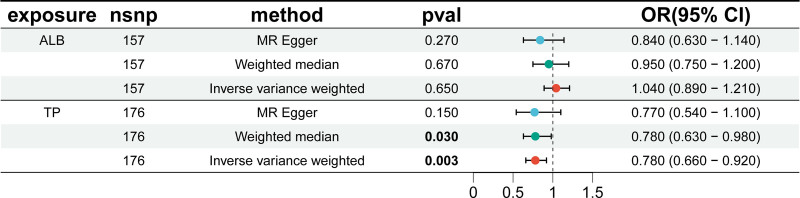
Mendelian randomization estimates of the causal effects of ALB and TP on CRC risk. ALB = albumin, CRC = colorectal cancer, TP = total protein.

Using SNPs significantly associated with ALB, IVW analysis showed no significant causal association between ALB and CRC (OR = 1.04, 95% CI = 0.89–1.21, *P* = .65), MR-Egger (0R = 0.84, 95% CI = 0.63–1.14, *P* = .27), or weighted median (OR = 0.95, 95% CI = 0.75–1.20, *P* = .67) (Fig. 5).

#### 3.4.2. There was significant causal association between TP and CRC

Using SNPs significantly associated with TP, IVW analyses showed a significant causal association between TP and CRC, with TP reducing the risk of CRC (OR = 0.78, 95% CI = 0.66–0.92, *P* = .003). MR-Egger (OR = 0.77, 95% CI = 0.54–1.20, *P* = .15) and the weighted median (OR = 0.78, 95% CI = 0.63–0.98, *P* = .029) showed similar results, although MR-Egger showed that the relationship was not statistically significant (Fig. 5).

## 4. Discussion

In this study, we used multiple MR methods to analyze GWAS data from the UK Biobank to explore the possible causal relationships between ALB, TP, and CRC. Our results demonstrated no significant causal relationship between ALB and CRC. There was a significant causal relationship between TP and CRC, with TP reducing the risk of CRC. These findings provide new insights into CRC prevention.

The causal relationship between ALB, TP, and CRC has not yet been fully investigated. Previous studies have explored the correlations between ALB, TP, and CRC. Studies on the relationship between ALB and CRC have reached mixed conclusions, and a prospective study based on a European population that included a case–control study of 256 patients with CRC found no significant correlation between ALB and CRC.^[13]^ A prospective UK BioBank-based study that investigated 375,693 participants showed that higher circulating levels of ALB were associated with a lower risk of CRC.^[11]^ Another Finnish-based study, which included 177 patients with bowel cancer and 288 age-, sex-, and region-matched control cases, showed that high ALB increases the risk of distal colon cancer.^[15]^ A previous MR study explored the relationship between ALB and CRC.^[9]^ A previous MR study extracted CRC data from Ishigaki et al.^[26]^ Disease group data were obtained from the BioBank Japan database for 7062 patients with CRC. Control data were obtained from the BioBank Japan database and several other Japanese databases, containing a total of 195,745 controls, and 13 SNPs were extracted. ALB data were extracted from an ALB GWAS of 102,223 East Asian participants,^[9,27]^ which yielded 17 SNPs, and analyses showed a significant causal association between ALB and CRC, with an OR of 0.75.

Few studies have investigated the association between TP and CRC. A case–control study based on a population-based serum bank established in Washington County, Maryland, which included 118 CRC patients and 118 controls, showed that TP was significantly lower in CRC patients than in controls.^[14]^ To our knowledge, there is a lack of studies on the causal relationship between ALB, TP, and CRC in European populations. Studying the causal relationship between ALB, TP, and CRC using MR will facilitate the early screening and prevention of the disease.

Because most observational studies were case–control studies, the temporal sequential relationship between exposure and outcome could not be ensured, and the possibility of reverse causality exists. In addition, studies on the relationship between ALB and CRC lack uniform conclusions owing to confounding factors and heterogeneity. To the best of our knowledge, studies on the causal relationship between TP and CRC are insufficient. RCTs are considered to have the highest level of evidence, apart from meta-analyses, for exploring causal relationships between exposures and outcomes, but are a huge investment in time, money, and manpower, and are not as practical as MR.

We analyzed whether there is a causal relationship between ALB, TP, and CRC using 3 different MR methods: IVW, MR-Egger, and weighted median. Genetic variants for ALB were obtained from the UK Biobank GWAS, which included 315,268 European participants, and SNPs significantly associated with ALB were extracted. CRC data were obtained from the most recent version of the FinnGen Consortium (version R9), which included 6509 CRC cases and 287,137 control cases. The results of the study suggested that there was no significant causal relationship between ALB and CRC (OR = 1.04, 95% CI = 0.89–1.21, *P* = .65). This result is inconsistent with that of the study by Lv et al,^[9]^ which may be due to the different ethnicities of the study population. The genetic variation in TP in this study was obtained from a UK Biobank study that included 314,921 Europeans and extracted SNPs that were significantly associated with TP. The findings suggested a significant causal relationship between TP and CRC (OR = 0.78, 95% CI = 0.66–0.92, *P* = .003). The risk of CRC was reduced by 22% for every 1-standard deviation increase in TP. The *P*-values of IVW and weighted median were all <.05. The IVW, MR-Egger, and weighted medians were in the same direction. IVW and MR-Egger showed significant heterogeneity, and we used IVW’s random effects model for statistics, which can minimize the effect of heterogeneity on the causality statistics. The leave-one-out test confirms that the causality statistical results are robust. The findings of previous studies showed that low TP was significantly associated with CRC,^[14]^ and high TP was significantly associated with a lower risk of CRC,^[11]^ which is in agreement with our view. A previous study found that high TP was associated with better survival in cancer-related malignancies,^[28]^ which is also consistent with our findings.

In this study, a 1-standard deviation increase in serum TP (approximately 5.3 g/L) was associated with a 22% reduced risk of CRC (OR = 0.78), which appears clinically meaningful given the typical serum protein range of 60 to 80 g/L. This effect size is consistent with prior observational findings. For example, Ko et al reported an increased colon cancer risk in individuals with lower TP levels (OR = 1.94 for lowest vs highest quartile).^[14]^ The potential mechanism between TP and CRC can be explained as follows. TP is a hepatic metabolite,^[11]^ and its decline is associated with reduced liver function, which has been shown to be associated with CRC.^[29]^ TP is a common indicator used to evaluate nutritional status, which is associated with the development of CRC.^[30]^ TP consists of several types of proteins, of which ALB and globulin are predominant; globulin includes α, β, and γ globulin; immunoglobulins belonging to the γ-globulin family can recognize and remove foreign molecules, and a decrease in globulin is detrimental to the removal of tumor components. Our study showed no significant causal relationship between ALB and CRC; therefore, it is possible that the causal relationship between TP and CRC was derived from globulin.

MR studies have several advantages. First, because genes are randomly assigned as they are passed from parent to offspring, MR exploits this principle to minimize confounding factor interference, and because there is a temporal sequence between genes and diseases, reverse causality can be avoided as much as possible.^[31]^ Second, we had large data sizes, using a GWAS of ALB containing 315,268 participants and a GWAS of TP containing 314,921 participants. The outcome date was a GWAS for CRC containing 6509 cases and 287,137 controls. Third, the GWAS of ALB, TP, and CRC were obtained from the UK Biobank and FinnGen Consortium, respectively, and the participants were of European ethnicity, which could reduce the bias caused by ethnic stratification. Fourth, our results show a causal relationship between TP and CRC, which will facilitate screening and early prevention of CRC. For patients with decreased TP, the focus should be on improving further screening for CRC and implementing regular follow-ups.

However, our study has some shortcomings. First, our GWAS data on exposure and outcome were mainly derived from a European sample; therefore, caution may be warranted in generalizing the findings of this study to various populations around the globe. Second, we only reported the causal relationship between TP and CRC, and the underlying mechanism still deserves further investigation. Third, the sample size, although substantial for detecting moderate to strong effects, may have limited statistical power to identify weaker associations, such as those potentially involving ALB. Consequently, we cannot rule out the possibility of type II errors (false negatives) for variables with very small effect sizes. Future validation with larger cohorts is warranted to explore these subtler relationships with greater confidence. Last, our study reported a causal relationship between TP and CRC, and its reverse causality will be explored in future studies.

## 5. Conclusion

Our MR findings suggest a causal relationship between TP and CRC, and no significant causal relationship between ALB and CRC. TP protects against CRC. When patients show a decrease in serum TP levels, colonoscopy and regular follow-up should be performed.

## Acknowledgments

We thank the participants and investigators of the UK Biobank and FinnGen consortium for sharing the data.

## Author contributions

**Data curation:** Jia Chen, Jiong Chen.

**Formal analysis:** Jia Chen, Jiong Chen.

**Funding acquisition:** Fulin Wang.

**Methodology:** Yong Kuang.

**Software:** Xin Zhong.

**Writing – original draft:** Jia Chen, Xin Zhong, Fulin Wang.

**Writing – review & editing:** Jia Chen, Yong Kuang.

## Supplementary Material


