# Supplementary material for: Causal relationship between albumin, total protein, and colorectal cancer risk: A 2-sample Mendelian randomization study
Source: Medicine (Baltimore). 2025 Oct 17;104(42):e45229. doi: 10.1097/MD.0000000000045229 (PMC12537268; doi:10.1097/MD.0000000000045229)
Supplement: Supplementary file 1 [file medi-104-e45229-s001.docx]

Supplementary Table 1

A

| SNP | EA | OA | Exposure | | | Outcome | | |
| --- | --- | --- | --- | --- | --- | --- | --- | --- |
|  |  |  | beta | se | pval | beta | se | pval |
| rs10004084 | C | T | 0.03 | 0.003 | 1.51E-19 | -0.011 | 0.027 | 0.693 |
| rs10042492 | T | C | -0.015 | 0.002 | 2.70E-09 | 0.001 | 0.018 | 0.971 |
| rs10143115 | A | G | -0.015 | 0.003 | 4.62E-09 | -0.012 | 0.018 | 0.525 |
| rs10213692 | C | T | 0.02 | 0.003 | 7.54E-12 | 0.02 | 0.024 | 0.41 |
| rs10236582 | C | T | -0.022 | 0.003 | 9.96E-16 | -0.029 | 0.022 | 0.179 |
| rs1030098 | C | T | 0.02 | 0.003 | 1.19E-15 | -0.01 | 0.019 | 0.618 |
| rs10419198 | T | C | -0.074 | 0.003 | 5.57E-149 | 0.018 | 0.021 | 0.377 |
| rs10431419 | A | C | -0.017 | 0.003 | 2.21E-08 | 0.002 | 0.02 | 0.935 |
| rs1043312 | G | T | 0.014 | 0.003 | 3.89E-08 | -0.02 | 0.019 | 0.288 |
| rs10456852 | T | C | 0.026 | 0.004 | 1.12E-12 | -0.054 | 0.031 | 0.082 |
| rs1079290 | A | T | 0.015 | 0.002 | 5.75E-10 | 0.018 | 0.018 | 0.32 |
| rs10793127 | A | G | 0.025 | 0.004 | 8.05E-09 | 0.036 | 0.029 | 0.223 |
| rs10863570 | C | T | 0.022 | 0.003 | 1.77E-15 | 0.015 | 0.02 | 0.446 |
| rs10919543 | G | A | -0.019 | 0.003 | 6.56E-13 | -0.02 | 0.021 | 0.334 |
| rs11012732 | G | A | -0.023 | 0.003 | 5.39E-19 | 0.014 | 0.02 | 0.464 |
| rs11057273 | C | T | -0.029 | 0.004 | 1.55E-11 | -0.026 | 0.029 | 0.365 |
| rs11074901 | A | G | 0.015 | 0.003 | 3.22E-08 | 0.018 | 0.019 | 0.353 |
| rs11078597 | C | T | 0.065 | 0.003 | 1.65E-95 | 0.025 | 0.023 | 0.278 |
| rs11088253 | T | C | 0.014 | 0.002 | 2.40E-08 | -0.026 | 0.018 | 0.158 |
| rs1110659 | C | T | 0.022 | 0.003 | 2.39E-14 | -0.031 | 0.023 | 0.179 |
| rs111443054 | TC AC AC A | T | -0.028 | 0.003 | 4.04E-19 | -0.682 | 1.077 | 0.526 |
| rs11217135 | T | C | -0.024 | 0.002 | 5.01E-23 | 0.01 | 0.018 | 0.596 |
| rs11264233 | A | G | 0.019 | 0.002 | 3.83E-15 | -0.006 | 0.018 | 0.721 |
| rs113177823 | A | G | 0.043 | 0.005 | 6.59E-15 | -0.014 | 0.038 | 0.72 |
| rs114949263 | C | T | 0.057 | 0.004 | 7.29E-48 | -0.042 | 0.034 | 0.217 |
| rs11589479 | A | G | 0.046 | 0.003 | 6.80E-43 | -0.034 | 0.026 | 0.187 |
| rs11609805 | A | G | -0.025 | 0.003 | 7.52E-19 | 0.019 | 0.021 | 0.367 |
| rs11656541 | C | G | 0.027 | 0.003 | 3.69E-25 | 0.024 | 0.018 | 0.182 |
| rs117127664 | T | C | 0.038 | 0.006 | 1.65E-09 | 0.022 | 0.035 | 0.531 |
| rs11736842 | T | A | 0.016 | 0.003 | 4.79E-10 | -0.005 | 0.018 | 0.78 |
| rs117820542 | G | C | -0.041 | 0.007 | 3.29E-08 | -0.046 | 0.077 | 0.556 |
| rs11895352 | T | C | -0.014 | 0.002 | 3.11E-08 | -0.026 | 0.018 | 0.157 |
| rs11928797 | A | C | 0.028 | 0.004 | 3.35E-13 | -0.026 | 0.028 | 0.354 |
| rs12215904 | T | C | -0.02 | 0.003 | 4.51E-10 | -0.032 | 0.025 | 0.199 |
| rs1229492 | C | T | -0.017 | 0.003 | 1.08E-09 | -0.002 | 0.021 | 0.942 |
| rs1229984 | C | T | 0.06 | 0.008 | 7.87E-13 | -0.146 | 0.126 | 0.248 |
| rs12377600 | G | A | -0.018 | 0.003 | 2.11E-12 | 0.001 | 0.019 | 0.973 |
| rs12563096 | A | G | 0.014 | 0.003 | 4.77E-08 | 0.019 | 0.019 | 0.327 |
| rs1260326 | C | T | -0.059 | 0.003 | 1.51E-121 | 0.037 | 0.019 | 0.05 |
| rs12710562 | G | A | 0.026 | 0.003 | 1.51E-16 | -0.041 | 0.025 | 0.097 |
| rs12815728 | C | T | 0.018 | 0.002 | 2.92E-13 | 0.003 | 0.018 | 0.861 |
| rs12881869 | T | C | -0.049 | 0.005 | 4.90E-25 | 0.015 | 0.031 | 0.617 |
| rs13107325 | T | C | -0.056 | 0.005 | 7.81E-33 | -0.066 | 0.076 | 0.383 |
| rs13108218 | G | A | -0.044 | 0.003 | 3.01E-67 | -0.041 | 0.019 | 0.034 |
| rs13111128 | A | G | -0.019 | 0.003 | 6.99E-12 | 0.013 | 0.019 | 0.496 |
| rs13389219 | T | C | -0.029 | 0.003 | 2.07E-31 | -0.041 | 0.019 | 0.032 |
| rs138833981 | G | C | -0.086 | 0.01 | 4.19E-18 | -0.031 | 0.097 | 0.746 |
| rs139278099 | T | C | 0.063 | 0.006 | 4.47E-24 | -0.039 | 0.083 | 0.637 |
| rs139974673 | C | T | 0.151 | 0.008 | 4.34E-82 | -0.19 | 0.126 | 0.131 |
| rs147651823 | A | C | -0.035 | 0.006 | 3.03E-09 | 0.083 | 0.044 | 0.059 |
| rs149092986 | C | T | -0.055 | 0.008 | 5.07E-11 | 0.133 | 0.063 | 0.036 |
| rs1497406 | G | A | 0.025 | 0.002 | 8.55E-24 | -0.01 | 0.019 | 0.615 |
| rs1500187 | G | A | -0.024 | 0.002 | 9.44E-22 | 0.002 | 0.018 | 0.894 |
| rs150783681 | C | G | -0.119 | 0.009 | 2.77E-42 | 0.054 | 0.071 | 0.447 |
| rs1532085 | G | A | 0.014 | 0.003 | 2.06E-08 | -0.009 | 0.018 | 0.63 |
| rs157936 | G | T | -0.019 | 0.003 | 1.61E-12 | 0.013 | 0.019 | 0.493 |
| rs1593357 | T | C | -0.02 | 0.003 | 1.18E-12 | -0.004 | 0.023 | 0.864 |
| rs16950612 | G | A | -0.025 | 0.004 | 1.10E-10 | -0.006 | 0.034 | 0.852 |
| rs17023530 | A | T | -0.038 | 0.006 | 2.53E-11 | 0.054 | 0.044 | 0.228 |
| rs17580 | A | T | 0.095 | 0.006 | 7.81E-62 | 0.066 | 0.094 | 0.485 |
| rs1782455 | A | G | 0.02 | 0.003 | 3.55E-09 | 0.037 | 0.024 | 0.12 |
| rs1791936 | A | G | 0.021 | 0.003 | 2.31E-17 | -0.016 | 0.019 | 0.395 |
| rs1801282 | G | C | -0.051 | 0.004 | 4.57E-41 | 0.041 | 0.024 | 0.091 |
| rs1880241 | G | A | 0.016 | 0.002 | 2.85E-10 | -0.002 | 0.019 | 0.91 |
| rs1886839 | G | A | 0.014 | 0.003 | 2.04E-08 | 0.014 | 0.018 | 0.451 |
| rs198426 | T | C | -0.017 | 0.003 | 8.75E-11 | -0.018 | 0.02 | 0.374 |
| rs1986133 | T | C | 0.016 | 0.003 | 1.32E-09 | 0.036 | 0.021 | 0.08 |
| rs1998528 | A | G | 0.015 | 0.003 | 3.98E-09 | 0.003 | 0.019 | 0.882 |
| rs2060658 | C | T | 0.017 | 0.002 | 2.16E-11 | 0.059 | 0.018 | 0.001 |
| rs2072442 | G | C | 0.015 | 0.002 | 5.51E-09 | -0.002 | 0.018 | 0.898 |
| rs2115868 | A | T | -0.024 | 0.003 | 3.03E-13 | -0.05 | 0.028 | 0.072 |
| rs2169387 | G | A | 0.058 | 0.004 | 1.40E-44 | 0.036 | 0.026 | 0.16 |
| rs2200061 | A | G | -0.017 | 0.003 | 4.33E-08 | 0.009 | 0.02 | 0.656 |
| rs2227827 | T | C | -0.051 | 0.006 | 5.99E-19 | 0.03 | 0.045 | 0.512 |
| rs2267867 | G | A | -0.016 | 0.003 | 2.75E-08 | -0.034 | 0.021 | 0.103 |
| rs2303695 | T | C | -0.026 | 0.003 | 2.81E-23 | -0.008 | 0.019 | 0.668 |
| rs2304130 | G | A | 0.027 | 0.004 | 7.30E-10 | 0.066 | 0.039 | 0.09 |
| rs234043 | C | T | 0.017 | 0.003 | 6.08E-10 | 0.032 | 0.021 | 0.126 |
| rs2638315 | C | G | 0.035 | 0.003 | 1.07E-28 | -0.002 | 0.023 | 0.947 |
| rs2702571 | A | T | 0.017 | 0.003 | 8.12E-11 | -0.037 | 0.02 | 0.063 |
| rs2710804 | C | T | -0.016 | 0.003 | 9.55E-11 | -0.019 | 0.019 | 0.318 |
| rs2785172 | A | G | -0.019 | 0.003 | 3.49E-14 | 0.011 | 0.019 | 0.546 |
| rs2820446 | G | C | -0.024 | 0.003 | 4.11E-19 | -0.005 | 0.02 | 0.788 |
| rs28601761 | G | C | -0.02 | 0.003 | 2.53E-15 | -0.04 | 0.018 | 0.03 |
| rs28687959 | T | C | 0.016 | 0.002 | 3.40E-11 | 0.005 | 0.018 | 0.781 |
| rs28688002 | A | T | 0.027 | 0.003 | 3.02E-20 | -0.04 | 0.02 | 0.046 |
| rs2869876 | A | C | 0.018 | 0.003 | 1.86E-08 | -0.015 | 0.022 | 0.478 |
| rs28929474 | T | C | 0.265 | 0.009 | 1.00E-200 | -0.014 | 0.065 | 0.828 |
| rs2972145 | C | T | 0.021 | 0.003 | 8.16E-17 | -0.002 | 0.019 | 0.909 |
| rs3099371 | T | C | 0.016 | 0.003 | 2.65E-10 | -0.012 | 0.019 | 0.52 |
| rs3184504 | C | T | 0.021 | 0.002 | 3.81E-18 | 0.054 | 0.018 | 0.004 |
| rs34562254 | A | G | -0.038 | 0.004 | 1.20E-19 | 0.043 | 0.03 | 0.153 |
| rs34931250 | T | C | 0.033 | 0.005 | 7.66E-11 | 0.077 | 0.037 | 0.038 |
| rs35123414 | C | T | 0.022 | 0.004 | 2.10E-09 | 0.039 | 0.024 | 0.099 |
| rs35676551 | A | C | -0.038 | 0.006 | 7.92E-12 | 0.042 | 0.036 | 0.237 |
| rs36090025 | C | A | 0.017 | 0.003 | 9.74E-11 | -0.01 | 0.023 | 0.675 |
| rs3740688 | T | G | 0.024 | 0.002 | 1.47E-22 | -0.003 | 0.018 | 0.855 |
| rs3768321 | T | G | -0.028 | 0.003 | 4.75E-20 | 0.047 | 0.025 | 0.057 |
| rs378740 | A | C | 0.016 | 0.003 | 1.78E-08 | 0.013 | 0.018 | 0.49 |
| rs3810484 | G | A | 0.015 | 0.002 | 2.43E-09 | -0.005 | 0.019 | 0.804 |
| rs390801 | C | T | 0.021 | 0.003 | 3.22E-14 | 0.003 | 0.02 | 0.887 |
| rs4327724 | T | C | 0.031 | 0.005 | 9.62E-12 | -0.091 | 0.03 | 0.002 |
| rs4410790 | C | T | 0.015 | 0.003 | 4.14E-09 | -0.025 | 0.019 | 0.193 |
| rs4458838 | A | G | -0.016 | 0.003 | 1.30E-09 | -0.008 | 0.019 | 0.687 |
| rs4499445 | T | G | -0.022 | 0.003 | 6.94E-17 | -0.003 | 0.021 | 0.89 |
| rs45439091 | T | G | 0.032 | 0.005 | 2.38E-11 | 0.106 | 0.054 | 0.051 |
| rs45512696 | T | C | 0.08 | 0.003 | 2.66E-135 | -0.003 | 0.025 | 0.89 |
| rs459193 | G | A | 0.023 | 0.003 | 1.50E-14 | 0.028 | 0.019 | 0.156 |
| rs4711399 | T | C | 0.022 | 0.003 | 1.29E-13 | 0.058 | 0.021 | 0.006 |
| rs473919 | G | C | -0.019 | 0.003 | 1.50E-09 | 0.019 | 0.022 | 0.401 |
| rs4782568 | G | C | -0.018 | 0.002 | 1.31E-13 | -0.028 | 0.018 | 0.119 |
| rs4790875 | T | C | -0.021 | 0.003 | 1.71E-16 | 0.02 | 0.019 | 0.274 |
| rs4804413 | T | C | 0.018 | 0.002 | 1.54E-12 | -0.008 | 0.018 | 0.666 |
| rs4805129 | C | T | -0.021 | 0.003 | 1.13E-16 | 0.048 | 0.019 | 0.014 |
| rs4805881 | C | A | -0.027 | 0.003 | 3.60E-25 | -0.001 | 0.019 | 0.946 |
| rs4833945 | C | T | 0.022 | 0.004 | 1.79E-08 | 0.026 | 0.027 | 0.336 |
| rs4946811 | C | A | 0.016 | 0.003 | 5.92E-10 | 0.005 | 0.018 | 0.775 |
| rs4970834 | T | C | -0.025 | 0.003 | 1.13E-15 | 0.012 | 0.023 | 0.613 |
| rs55696240 | A | G | 0.017 | 0.003 | 2.42E-11 | -0.022 | 0.019 | 0.233 |
| rs55722786 | T | G | 0.028 | 0.003 | 7.79E-25 | -0.021 | 0.022 | 0.332 |
| rs55724869 | C | G | 0.014 | 0.003 | 1.46E-08 | -0.013 | 0.019 | 0.485 |
| rs55846720 | A | G | -0.015 | 0.002 | 2.40E-09 | -0.016 | 0.018 | 0.395 |
| rs56094005 | G | A | -0.047 | 0.006 | 1.08E-14 | -0.072 | 0.043 | 0.089 |
| rs56188865 | C | T | 0.016 | 0.003 | 2.07E-10 | 0.006 | 0.019 | 0.761 |
| rs58558667 | T | G | 0.023 | 0.003 | 1.81E-19 | -0.015 | 0.018 | 0.427 |
| rs58579887 | C | T | 0.018 | 0.003 | 3.43E-13 | 0.034 | 0.019 | 0.07 |
| rs59431480 | G | C | 0.086 | 0.013 | 1.60E-11 | -0.351 | 0.144 | 0.015 |
| rs59916403 | T | G | -0.024 | 0.003 | 3.99E-21 | -0.045 | 0.018 | 0.013 |
| rs6031847 | T | C | -0.021 | 0.003 | 1.88E-14 | 0.009 | 0.021 | 0.68 |
| rs60644673 | T | G | -0.018 | 0.003 | 1.92E-08 | 0.024 | 0.022 | 0.262 |
| rs61552236 | ACT | A | -0.016 | 0.003 | 6.64E-09 | 0.012 | 0.02 | 0.561 |
| rs61817641 | T | C | -0.018 | 0.003 | 6.01E-11 | -0.018 | 0.021 | 0.383 |
| rs61983272 | C | G | -0.033 | 0.003 | 7.26E-31 | -0.009 | 0.02 | 0.631 |
| rs62053895 | A | G | -0.014 | 0.003 | 3.83E-08 | 0.015 | 0.019 | 0.423 |
| rs631695 | G | T | 0.015 | 0.002 | 5.28E-10 | 0.012 | 0.018 | 0.526 |
| rs6490409 | G | A | -0.019 | 0.004 | 3.17E-08 | -0.013 | 0.022 | 0.554 |
| rs6549406 | G | A | -0.019 | 0.003 | 1.00E-11 | 0.019 | 0.019 | 0.33 |
| rs6567095 | A | G | 0.019 | 0.002 | 5.46E-15 | -0.014 | 0.018 | 0.435 |
| rs667172 | A | G | 0.019 | 0.003 | 3.81E-12 | 0.015 | 0.02 | 0.471 |
| rs6693993 | C | G | 0.024 | 0.002 | 1.57E-21 | 0.006 | 0.018 | 0.744 |
| rs6734238 | G | A | -0.016 | 0.003 | 1.74E-10 | 0.017 | 0.02 | 0.391 |
| rs673751 | C | A | -0.015 | 0.003 | 6.77E-09 | 0.013 | 0.019 | 0.495 |
| rs67694436 | T | C | 0.015 | 0.003 | 5.00E-09 | -0.008 | 0.019 | 0.665 |
| rs6793835 | A | G | 0.016 | 0.003 | 4.69E-09 | -0.021 | 0.022 | 0.349 |
| rs6794370 | C | A | -0.019 | 0.003 | 7.27E-10 | 0.002 | 0.021 | 0.937 |
| rs6860245 | C | G | -0.021 | 0.003 | 6.54E-14 | 0.035 | 0.025 | 0.164 |
| rs6871748 | C | T | 0.015 | 0.003 | 3.36E-08 | 0.027 | 0.019 | 0.168 |
| rs6897617 | A | G | 0.018 | 0.003 | 1.84E-11 | -0.001 | 0.019 | 0.954 |
| rs6912315 | T | C | 0.031 | 0.005 | 1.16E-08 | -0.11 | 0.068 | 0.106 |
| rs6970593 | A | G | -0.026 | 0.002 | 5.84E-25 | -0.042 | 0.018 | 0.024 |
| rs7031621 | A | G | -0.014 | 0.002 | 5.18E-09 | 0.039 | 0.018 | 0.031 |
| rs72631343 | G | C | 0.022 | 0.004 | 4.63E-09 | 0.016 | 0.026 | 0.528 |
| rs72789541 | A | T | 0.018 | 0.003 | 1.52E-11 | -0.023 | 0.019 | 0.231 |
| rs72793380 | A | C | 0.022 | 0.004 | 2.47E-08 | -0.004 | 0.032 | 0.907 |
| rs72818989 | C | T | 0.016 | 0.002 | 2.21E-10 | 0.003 | 0.018 | 0.881 |
| rs72959041 | A | G | 0.051 | 0.006 | 2.94E-19 | 0.053 | 0.038 | 0.161 |
| rs73038384 | T | C | -0.052 | 0.007 | 1.18E-13 | -0.03 | 0.045 | 0.503 |
| rs73234873 | T | C | -0.015 | 0.003 | 3.22E-08 | -0.018 | 0.022 | 0.422 |
| rs7366884 | C | T | 0.019 | 0.003 | 1.56E-11 | 0.011 | 0.021 | 0.613 |
| rs7402977 | A | G | -0.016 | 0.003 | 6.44E-09 | -0.049 | 0.02 | 0.016 |
| rs74538877 | C | G | 0.031 | 0.005 | 1.51E-08 | 0.069 | 0.038 | 0.066 |
| rs74780677 | G | A | 0.093 | 0.01 | 3.21E-20 | 0.022 | 0.035 | 0.529 |
| rs7591567 | C | T | 0.016 | 0.003 | 7.66E-09 | -0.009 | 0.02 | 0.652 |
| rs76895963 | G | T | -0.071 | 0.009 | 4.80E-14 | 0.091 | 0.054 | 0.089 |
| rs7731045 | C | T | -0.014 | 0.003 | 2.23E-08 | -0.02 | 0.018 | 0.292 |
| rs77542162 | G | A | -0.193 | 0.008 | 1.77E-122 | -0.046 | 0.109 | 0.671 |
| rs77849807 | G | A | 0.114 | 0.01 | 1.31E-30 | -0.036 | 0.059 | 0.541 |
| rs78444263 | T | C | -0.035 | 0.005 | 2.11E-13 | -0.025 | 0.029 | 0.397 |
| rs78961851 | C | T | -0.025 | 0.004 | 1.18E-09 | -0.035 | 0.035 | 0.31 |
| rs79295634 | G | A | 0.031 | 0.005 | 2.22E-10 | 0.008 | 0.034 | 0.803 |
| rs800545 | G | A | -0.016 | 0.003 | 1.15E-08 | 0.018 | 0.02 | 0.385 |
| rs8041057 | T | C | -0.023 | 0.003 | 1.45E-17 | -0.062 | 0.019 | 0.001 |
| rs8072215 | G | A | -0.034 | 0.003 | 2.96E-31 | -0.006 | 0.021 | 0.764 |
| rs854796 | A | G | 0.015 | 0.003 | 5.59E-09 | 0.00E+00 | 0.02 | 0.999 |
| rs872926 | A | G | -0.036 | 0.003 | 4.05E-32 | 0.069 | 0.024 | 0.003 |
| rs879620 | T | C | -0.014 | 0.003 | 2.33E-08 | 0.002 | 0.018 | 0.907 |
| rs900400 | C | T | 0.023 | 0.003 | 3.37E-19 | 0.012 | 0.02 | 0.531 |
| rs9265945 | G | A | -0.023 | 0.003 | 6.83E-21 | -0.029 | 0.019 | 0.117 |
| rs930340 | A | G | -0.019 | 0.003 | 2.32E-09 | 0 | 0.024 | 0.996 |
| rs9391997 | G | A | -0.016 | 0.002 | 5.93E-11 | 0.024 | 0.018 | 0.194 |
| rs9638180 | G | A | -0.024 | 0.003 | 2.47E-15 | 0.019 | 0.024 | 0.41 |
| rs9917677 | C | T | -0.016 | 0.003 | 4.25E-08 | -0.002 | 0.022 | 0.929 |
| rs992367 | G | A | 0.015 | 0.003 | 1.04E-09 | 0.028 | 0.019 | 0.152 |
| rs9976946 | T | C | 0.039 | 0.006 | 1.47E-09 | 0.015 | 0.072 | 0.835 |

B

| SNP | EA | OA | Exposure | | | Outcome | | |
| --- | --- | --- | --- | --- | --- | --- | --- | --- |
|  |  |  | beta | se | pval | beta | se | pval |
| rs10004084 | C | T | 0.035 | 0.003 | 1.54E-25 | -0.011 | 0.027 | 0.693 |
| rs10065637 | T | C | -0.04 | 0.003 | 1.36E-37 | 0.012 | 0.026 | 0.662 |
| rs10069690 | T | C | -0.022 | 0.003 | 2.97E-14 | -0.08 | 0.02 | 0 |
| rs10111287 | T | C | 0.019 | 0.003 | 2.05E-11 | -0.014 | 0.018 | 0.457 |
| rs10160596 | A | G | -0.02 | 0.003 | 1.22E-12 | -0.006 | 0.023 | 0.802 |
| rs1020048 | C | A | -0.027 | 0.003 | 1.38E-16 | -0.001 | 0.024 | 0.964 |
| rs10419198 | T | C | -0.075 | 0.003 | 8.85E-147 | 0.018 | 0.021 | 0.377 |
| rs10444863 | C | T | -0.028 | 0.003 | 5.49E-20 | 0.07 | 0.024 | 0.003 |
| rs1047891 | A | C | -0.026 | 0.003 | 1.85E-21 | 0.021 | 0.019 | 0.27 |
| rs10745495 | G | C | -0.017 | 0.003 | 7.80E-09 | 0.002 | 0.02 | 0.935 |
| rs10750400 | G | A | 0.018 | 0.003 | 2.20E-13 | 0.044 | 0.018 | 0.017 |
| rs10846690 | C | T | 0.026 | 0.004 | 1.01E-13 | 0.036 | 0.021 | 0.086 |
| rs10882100 | G | C | 0.015 | 0.003 | 1.69E-09 | -0.015 | 0.018 | 0.411 |
| rs10887917 | C | G | 0.018 | 0.003 | 1.87E-12 | -0.004 | 0.019 | 0.82 |
| rs10898811 | A | T | -0.018 | 0.003 | 2.75E-12 | -0.024 | 0.018 | 0.189 |
| rs11099882 | C | T | -0.021 | 0.003 | 9.21E-15 | -0.011 | 0.018 | 0.536 |
| rs11102009 | T | C | -0.02 | 0.003 | 1.00E-14 | 0.04 | 0.018 | 0.03 |
| rs11118320 | G | C | 0.017 | 0.003 | 9.28E-12 | 0.009 | 0.018 | 0.607 |
| rs11128592 | G | T | 0.026 | 0.004 | 1.18E-12 | -0.06 | 0.031 | 0.053 |
| rs11138299 | A | C | -0.028 | 0.005 | 1.05E-09 | 0.049 | 0.037 | 0.189 |
| rs11217863 | A | G | 0.023 | 0.004 | 3.07E-09 | -0.013 | 0.027 | 0.636 |
| rs112733823 | T | C | 0.033 | 0.004 | 1.07E-17 | -0.065 | 0.022 | 0.003 |
| rs113810201 | G | A | 0.025 | 0.004 | 1.79E-10 | -0.033 | 0.027 | 0.225 |
| rs114310991 | A | G | -0.115 | 0.01 | 4.10E-28 | -0.111 | 0.296 | 0.706 |
| rs11447452 | CT | C | 0.024 | 0.003 | 2.05E-14 | 0.019 | 0.021 | 0.367 |
| rs114949263 | C | T | 0.03 | 0.004 | 3.60E-14 | -0.042 | 0.034 | 0.217 |
| rs11594976 | C | T | -0.014 | 0.003 | 2.18E-08 | -0.024 | 0.018 | 0.188 |
| rs11620783 | T | C | 0.018 | 0.003 | 1.96E-12 | -0.037 | 0.019 | 0.052 |
| rs11711621 | T | C | 0.018 | 0.003 | 3.94E-10 | -0.049 | 0.02 | 0.016 |
| rs118083884 | A | G | -0.123 | 0.011 | 1.01E-29 | -0.032 | 0.107 | 0.763 |
| rs11879090 | T | C | -0.014 | 0.003 | 1.49E-08 | 0.011 | 0.018 | 0.541 |
| rs1196837 | G | A | -0.017 | 0.003 | 1.63E-11 | 0.038 | 0.018 | 0.039 |
| rs12142550 | T | C | 0.018 | 0.003 | 2.39E-10 | 0.022 | 0.021 | 0.295 |
| rs12363520 | A | T | -0.017 | 0.003 | 1.03E-08 | 0.015 | 0.021 | 0.489 |
| rs12377600 | G | A | -0.017 | 0.003 | 1.32E-10 | 0.001 | 0.019 | 0.973 |
| rs12386224 | C | G | 0.015 | 0.003 | 3.06E-08 | 0.002 | 0.019 | 0.918 |
| rs12434109 | C | T | -0.016 | 0.003 | 1.31E-09 | -0.006 | 0.019 | 0.769 |
| rs12453969 | A | G | 0.022 | 0.003 | 2.69E-16 | -0.03 | 0.02 | 0.13 |
| rs12505932 | G | T | 0.015 | 0.003 | 1.09E-08 | 0.039 | 0.019 | 0.039 |
| rs12544863 | T | C | -0.021 | 0.003 | 1.17E-15 | -0.001 | 0.019 | 0.971 |
| rs1260326 | C | T | -0.042 | 0.003 | 3.42E-61 | 0.037 | 0.019 | 0.05 |
| rs1271309 | G | A | 0.019 | 0.003 | 3.49E-08 | 0.028 | 0.029 | 0.35 |
| rs12722497 | A | C | 0.037 | 0.004 | 2.81E-16 | -0.025 | 0.036 | 0.487 |
| rs12881869 | T | C | -0.032 | 0.005 | 4.23E-11 | 0.015 | 0.031 | 0.617 |
| rs13005282 | G | A | -0.028 | 0.003 | 3.44E-27 | 0.063 | 0.019 | 0.001 |
| rs13020178 | A | G | 0.014 | 0.003 | 1.41E-08 | 0.005 | 0.019 | 0.806 |
| rs13108218 | G | A | -0.035 | 0.003 | 4.98E-41 | -0.041 | 0.019 | 0.034 |
| rs13266875 | T | A | -0.028 | 0.004 | 2.56E-11 | -0.009 | 0.027 | 0.75 |
| rs13278421 | G | A | -0.019 | 0.004 | 4.64E-08 | -0.004 | 0.027 | 0.87 |
| rs13322435 | G | A | 0.022 | 0.003 | 2.17E-17 | 0.009 | 0.019 | 0.628 |
| rs13380871 | C | T | 0.057 | 0.007 | 6.44E-17 | -0.015 | 0.045 | 0.742 |
| rs13425999 | T | C | -0.035 | 0.005 | 9.86E-13 | 0.036 | 0.042 | 0.395 |
| rs138833981 | G | C | -0.074 | 0.01 | 2.88E-13 | -0.031 | 0.097 | 0.746 |
| rs139974673 | C | T | 0.104 | 0.008 | 4.45E-38 | -0.19 | 0.126 | 0.131 |
| rs140118846 | A | G | -0.074 | 0.009 | 2.73E-16 | -0.054 | 0.101 | 0.593 |
| rs1448187 | C | T | -0.04 | 0.003 | 6.13E-41 | -0.011 | 0.021 | 0.608 |
| rs1458019 | G | A | 0.029 | 0.003 | 8.22E-24 | -0.002 | 0.02 | 0.925 |
| rs148393876 | A | C | -0.052 | 0.006 | 8.95E-20 | 0.025 | 0.042 | 0.544 |
| rs149914551 | A | C | -0.045 | 0.007 | 2.92E-11 | 0.151 | 0.058 | 0.009 |
| rs1561721 | C | T | -0.018 | 0.003 | 3.84E-10 | -0.002 | 0.022 | 0.91 |
| rs1571878 | T | C | -0.017 | 0.003 | 1.39E-11 | 0.047 | 0.018 | 0.011 |
| rs157936 | G | T | -0.021 | 0.003 | 7.98E-14 | 0.013 | 0.019 | 0.493 |
| rs1611236 | A | G | -0.038 | 0.003 | 4.91E-46 | 0.019 | 0.022 | 0.387 |
| rs1657792 | C | T | 0.019 | 0.003 | 3.45E-14 | 0.005 | 0.019 | 0.795 |
| rs16826069 | G | A | -0.023 | 0.003 | 1.63E-13 | 0.043 | 0.023 | 0.063 |
| rs1689789 | A | G | -0.019 | 0.003 | 1.41E-13 | -0.021 | 0.02 | 0.282 |
| rs17090693 | C | T | -0.018 | 0.003 | 2.12E-10 | -0.017 | 0.021 | 0.41 |
| rs17711850 | C | T | 0.028 | 0.003 | 7.25E-27 | -0.047 | 0.021 | 0.024 |
| rs17764730 | T | C | -0.022 | 0.003 | 1.58E-14 | 0.035 | 0.025 | 0.164 |
| rs1801282 | G | C | -0.041 | 0.004 | 3.20E-26 | 0.041 | 0.024 | 0.091 |
| rs181242111 | A | G | 0.035 | 0.004 | 4.04E-17 | -0.023 | 0.032 | 0.47 |
| rs1975161 | C | T | -0.019 | 0.003 | 5.02E-13 | 0.015 | 0.02 | 0.463 |
| rs2014842 | A | G | 0.019 | 0.003 | 1.02E-08 | -0.035 | 0.02 | 0.085 |
| rs201822981 | AT | A | -0.021 | 0.003 | 2.46E-15 | -0.324 | 0.19 | 0.088 |
| rs2068888 | A | G | -0.016 | 0.003 | 6.45E-11 | 0.021 | 0.018 | 0.249 |
| rs2236295 | T | G | -0.017 | 0.003 | 6.91E-11 | -0.003 | 0.019 | 0.866 |
| rs2273215 | A | G | -0.014 | 0.003 | 2.48E-08 | 0.007 | 0.019 | 0.713 |
| rs2283620 | G | C | -0.016 | 0.003 | 4.73E-09 | -0.017 | 0.019 | 0.357 |
| rs2293445 | A | G | -0.016 | 0.003 | 2.49E-10 | -0.017 | 0.018 | 0.347 |
| rs2371108 | T | G | 0.014 | 0.003 | 2.74E-08 | -0.013 | 0.019 | 0.474 |
| rs2396746 | T | C | 0.017 | 0.003 | 5.87E-11 | 0.031 | 0.019 | 0.102 |
| rs2412974 | T | C | -0.027 | 0.003 | 3.33E-25 | 0.041 | 0.018 | 0.026 |
| rs2442517 | G | A | -0.014 | 0.003 | 2.68E-08 | -0.02 | 0.018 | 0.27 |
| rs2466574 | G | A | 0.018 | 0.003 | 1.58E-10 | 0.018 | 0.021 | 0.388 |
| rs2516655 | A | G | -0.033 | 0.003 | 3.24E-36 | 0.039 | 0.019 | 0.042 |
| rs2518710 | G | C | -0.026 | 0.005 | 2.78E-08 | -0.065 | 0.04 | 0.102 |
| rs2532387 | T | G | -0.035 | 0.003 | 8.66E-30 | -0.058 | 0.032 | 0.075 |
| rs2549508 | G | A | 0.025 | 0.004 | 3.25E-11 | -0.008 | 0.026 | 0.768 |
| rs2681416 | A | G | 0.015 | 0.003 | 1.57E-08 | 0.024 | 0.021 | 0.251 |
| rs2720660 | G | A | -0.017 | 0.003 | 1.16E-10 | -0.02 | 0.02 | 0.296 |
| rs2774947 | T | C | 0.036 | 0.004 | 4.28E-16 | -0.045 | 0.03 | 0.13 |
| rs28368955 | T | C | -0.024 | 0.003 | 3.48E-12 | -0.014 | 0.024 | 0.571 |
| rs28499538 | C | A | -0.016 | 0.003 | 1.10E-08 | 0.001 | 0.019 | 0.941 |
| rs28505206 | G | A | 0.03 | 0.004 | 4.08E-16 | -0.043 | 0.03 | 0.154 |
| rs28929474 | T | C | 0.139 | 0.009 | 6.59E-55 | -0.014 | 0.065 | 0.828 |
| rs2972156 | G | C | 0.019 | 0.003 | 7.79E-13 | -0.003 | 0.019 | 0.874 |
| rs303929 | C | G | -0.015 | 0.003 | 4.37E-08 | -0.036 | 0.02 | 0.067 |
| rs3087243 | A | G | -0.02 | 0.003 | 2.65E-15 | 0.007 | 0.019 | 0.709 |
| rs34215892 | A | G | -0.043 | 0.008 | 1.72E-08 | 0.004 | 0.056 | 0.94 |
| rs34322 | C | T | -0.017 | 0.003 | 1.53E-11 | 0.018 | 0.019 | 0.335 |
| rs34562254 | A | G | 0.118 | 0.004 | 1.33E-170 | 0.043 | 0.03 | 0.153 |
| rs34592828 | A | G | -0.055 | 0.006 | 5.40E-20 | 0.018 | 0.046 | 0.705 |
| rs34642860 | T | C | 0.015 | 0.003 | 2.30E-08 | -0.049 | 0.021 | 0.021 |
| rs34936565 | TAC | T | -0.024 | 0.003 | 1.21E-14 | 0.695 | 0.48 | 0.148 |
| rs35049983 | A | G | -0.017 | 0.003 | 3.90E-10 | 0.014 | 0.02 | 0.491 |
| rs350822 | C | T | 0.023 | 0.003 | 5.62E-15 | 0.016 | 0.021 | 0.458 |
| rs352139 | C | T | 0.015 | 0.003 | 9.34E-09 | 0.017 | 0.018 | 0.354 |
| rs35986445 | C | T | -0.021 | 0.003 | 6.79E-11 | -0.007 | 0.024 | 0.77 |
| rs35996865 | G | T | -0.02 | 0.003 | 1.75E-10 | 0.021 | 0.025 | 0.393 |
| rs374039502 | A | T | 0.107 | 0.009 | 3.42E-30 | -0.085 | 0.104 | 0.416 |
| rs3740688 | T | G | 0.018 | 0.003 | 3.65E-12 | -0.003 | 0.018 | 0.855 |
| rs3755172 | C | T | 0.024 | 0.003 | 3.22E-12 | -0.009 | 0.028 | 0.744 |
| rs3756772 | T | C | 0.023 | 0.003 | 1.64E-18 | 0.003 | 0.018 | 0.859 |
| rs3781351 | A | T | 0.014 | 0.003 | 3.43E-08 | -0.014 | 0.019 | 0.453 |
| rs3790315 | T | C | -0.016 | 0.003 | 4.76E-10 | 0.014 | 0.018 | 0.441 |
| rs3798556 | C | G | -0.018 | 0.003 | 2.49E-08 | -0.037 | 0.022 | 0.091 |
| rs3803286 | G | A | -0.048 | 0.003 | 1.58E-72 | 0.016 | 0.019 | 0.389 |
| rs3803800 | G | A | -0.043 | 0.003 | 2.62E-44 | 0.012 | 0.021 | 0.56 |
| rs3810504 | A | G | 0.026 | 0.004 | 9.15E-12 | 0.04 | 0.027 | 0.145 |
| rs3811647 | A | G | 0.019 | 0.003 | 2.21E-13 | 0.012 | 0.02 | 0.524 |
| rs3811671 | A | C | -0.018 | 0.003 | 1.10E-08 | 0.02 | 0.021 | 0.337 |
| rs3812207 | A | G | -0.015 | 0.003 | 1.20E-08 | 0.004 | 0.018 | 0.838 |
| rs38246 | C | T | -0.018 | 0.003 | 4.33E-10 | -0.033 | 0.022 | 0.129 |
| rs3849768 | A | C | 0.02 | 0.003 | 8.48E-11 | -0.013 | 0.023 | 0.561 |
| rs3870401 | C | G | -0.016 | 0.003 | 8.36E-09 | 0.035 | 0.019 | 0.063 |
| rs3891176 | A | C | -0.075 | 0.003 | 1.10E-136 | 0.009 | 0.026 | 0.735 |
| rs41444548 | G | C | 0.046 | 0.005 | 1.33E-21 | -0.005 | 0.036 | 0.895 |
| rs42032 | A | G | -0.016 | 0.003 | 1.06E-08 | 0.036 | 0.021 | 0.082 |
| rs4482257 | G | C | -0.016 | 0.003 | 3.22E-08 | -0.003 | 0.02 | 0.869 |
| rs4649017 | C | T | 0.014 | 0.003 | 3.59E-08 | -0.003 | 0.019 | 0.851 |
| rs4729594 | C | T | -0.015 | 0.003 | 6.39E-09 | -0.041 | 0.018 | 0.026 |
| rs4790877 | A | G | -0.033 | 0.003 | 9.56E-38 | 0.025 | 0.018 | 0.178 |
| rs4805881 | C | A | -0.016 | 0.003 | 1.70E-09 | -0.001 | 0.019 | 0.946 |
| rs4845107 | G | A | -0.021 | 0.004 | 7.22E-09 | -0.026 | 0.025 | 0.302 |
| rs4852257 | G | T | 0.015 | 0.003 | 9.15E-09 | 0.059 | 0.018 | 0.001 |
| rs4938496 | G | A | -0.029 | 0.003 | 2.16E-26 | -0.039 | 0.019 | 0.043 |
| rs4940573 | G | T | 0.04 | 0.004 | 6.43E-27 | 0.028 | 0.023 | 0.234 |
| rs5001409 | C | A | -0.022 | 0.003 | 5.27E-18 | 0.015 | 0.018 | 0.406 |
| rs55772024 | A | G | -0.018 | 0.003 | 1.12E-09 | -0.007 | 0.019 | 0.718 |
| rs55844407 | T | C | -0.019 | 0.003 | 1.62E-09 | 0.008 | 0.021 | 0.722 |
| rs55865752 | A | G | 0.017 | 0.003 | 1.80E-08 | -0.03 | 0.023 | 0.185 |
| rs56031211 | A | G | -0.018 | 0.003 | 2.22E-08 | -0.019 | 0.023 | 0.418 |
| rs567743 | G | A | 0.017 | 0.003 | 9.65E-10 | -0.042 | 0.02 | 0.036 |
| rs58432776 | A | C | 0.021 | 0.003 | 1.18E-15 | -0.022 | 0.019 | 0.247 |
| rs58895965 | A | C | 0.049 | 0.003 | 5.08E-49 | -0.004 | 0.025 | 0.877 |
| rs59418918 | C | A | 0.014 | 0.003 | 1.94E-08 | -0.01 | 0.018 | 0.594 |
| rs59441438 | G | T | -0.024 | 0.004 | 3.73E-10 | -0.013 | 0.033 | 0.685 |
| rs61237993 | A | G | -0.022 | 0.004 | 8.40E-09 | 0.131 | 0.026 | 0 |
| rs61830291 | C | A | 0.03 | 0.004 | 1.72E-12 | -0.012 | 0.028 | 0.671 |
| rs62045817 | T | C | -0.025 | 0.004 | 2.22E-12 | 0.037 | 0.027 | 0.181 |
| rs62131205 | C | G | -0.04 | 0.005 | 2.81E-17 | 0.032 | 0.03 | 0.284 |
| rs62441844 | T | C | 0.018 | 0.003 | 1.12E-08 | -0.036 | 0.022 | 0.109 |
| rs631864 | C | T | -0.026 | 0.003 | 4.60E-24 | -0.001 | 0.018 | 0.968 |
| rs6447324 | T | G | -0.022 | 0.003 | 5.37E-12 | 0.048 | 0.02 | 0.018 |
| rs6557616 | C | G | 0.022 | 0.003 | 1.34E-12 | 0.015 | 0.021 | 0.486 |
| rs6600250 | T | C | -0.034 | 0.003 | 5.49E-42 | -0.003 | 0.018 | 0.85 |
| rs66477686 | C | T | -0.025 | 0.003 | 2.91E-19 | -0.013 | 0.021 | 0.534 |
| rs6665912 | C | T | 0.029 | 0.003 | 5.32E-22 | -0.014 | 0.021 | 0.511 |
| rs6720027 | T | C | 0.016 | 0.003 | 1.94E-09 | -0.044 | 0.02 | 0.029 |
| rs679574 | G | C | 0.038 | 0.003 | 4.23E-50 | 0.025 | 0.019 | 0.186 |
| rs6833591 | G | A | -0.018 | 0.003 | 8.02E-12 | -0.005 | 0.022 | 0.835 |
| rs6903640 | C | T | -0.021 | 0.003 | 2.90E-16 | -0.009 | 0.018 | 0.616 |
| rs6924387 | G | A | 0.025 | 0.003 | 1.35E-22 | -0.01 | 0.018 | 0.583 |
| rs6942338 | T | C | 0.047 | 0.005 | 9.06E-25 | 0.042 | 0.026 | 0.108 |
| rs7005025 | C | A | -0.018 | 0.003 | 8.58E-12 | 0.006 | 0.019 | 0.747 |
| rs71393469 | A | C | -0.029 | 0.004 | 1.37E-12 | -0.002 | 0.037 | 0.964 |
| rs7140 | A | C | -0.018 | 0.003 | 1.70E-11 | 0.006 | 0.019 | 0.756 |
| rs71423603 | T | C | -0.021 | 0.004 | 3.40E-08 | 0.059 | 0.034 | 0.088 |
| rs71610255 | C | A | -0.03 | 0.004 | 1.17E-15 | -0.025 | 0.023 | 0.283 |
| rs7197422 | G | C | 0.016 | 0.003 | 1.52E-10 | 0.025 | 0.019 | 0.179 |
| rs7228151 | C | T | 0.02 | 0.003 | 2.18E-10 | 0.015 | 0.021 | 0.471 |
| rs72665951 | T | C | 0.017 | 0.003 | 1.06E-10 | -0.007 | 0.019 | 0.694 |
| rs72704062 | G | C | -0.095 | 0.008 | 1.87E-31 | 0.023 | 0.041 | 0.578 |
| rs72799341 | A | G | 0.021 | 0.003 | 2.67E-13 | 0.012 | 0.02 | 0.565 |
| rs72832890 | G | A | 0.026 | 0.004 | 1.71E-09 | -0.017 | 0.029 | 0.545 |
| rs72835315 | C | T | 0.044 | 0.006 | 3.17E-15 | 0.078 | 0.029 | 0.007 |
| rs7287486 | T | G | -0.062 | 0.008 | 3.66E-14 | 0.057 | 0.059 | 0.331 |
| rs7295713 | T | C | -0.014 | 0.003 | 3.58E-08 | 0.005 | 0.018 | 0.805 |
| rs7304603 | C | T | -0.014 | 0.003 | 1.31E-08 | -0.028 | 0.019 | 0.137 |
| rs7421377 | A | C | 0.02 | 0.003 | 1.31E-14 | -0.008 | 0.02 | 0.695 |
| rs74922662 | A | G | 0.034 | 0.006 | 1.10E-08 | -0.072 | 0.052 | 0.168 |
| rs7502910 | G | A | 0.025 | 0.003 | 2.86E-23 | -0.006 | 0.018 | 0.739 |
| rs750598 | A | G | -0.022 | 0.003 | 3.56E-17 | -0.011 | 0.019 | 0.551 |
| rs75328154 | T | C | -0.018 | 0.003 | 3.21E-10 | 0.056 | 0.022 | 0.012 |
| rs75478023 | C | T | -0.036 | 0.005 | 4.21E-13 | -0.023 | 0.027 | 0.397 |
| rs75899192 | C | G | -0.032 | 0.005 | 3.29E-09 | 0.013 | 0.035 | 0.714 |
| rs76428106 | C | T | 0.13 | 0.011 | 1.43E-29 | -0.174 | 0.082 | 0.033 |
| rs7659414 | C | A | -0.018 | 0.003 | 5.49E-13 | 0.007 | 0.019 | 0.694 |
| rs7676237 | A | G | 0.015 | 0.003 | 4.06E-08 | 0.002 | 0.019 | 0.91 |
| rs76895963 | G | T | -0.077 | 0.01 | 1.01E-15 | 0.091 | 0.054 | 0.089 |
| rs77542162 | G | A | -0.097 | 0.008 | 9.69E-31 | -0.046 | 0.109 | 0.671 |
| rs7774393 | G | C | 0.021 | 0.003 | 2.11E-14 | -0.059 | 0.019 | 0.002 |
| rs77849807 | G | A | 0.126 | 0.01 | 1.18E-35 | -0.036 | 0.059 | 0.541 |
| rs7821812 | C | G | -0.037 | 0.003 | 1.13E-32 | 0.001 | 0.025 | 0.96 |
| rs78484938 | A | C | -0.037 | 0.007 | 3.77E-08 | 0.165 | 0.094 | 0.078 |
| rs7942247 | A | G | -0.017 | 0.003 | 1.07E-11 | 0.001 | 0.018 | 0.97 |
| rs7966399 | G | A | 0.02 | 0.003 | 1.77E-12 | -0.011 | 0.02 | 0.583 |
| rs7995886 | T | C | 0.016 | 0.003 | 1.08E-09 | 0.005 | 0.018 | 0.772 |
| rs8092360 | T | C | -0.021 | 0.003 | 4.36E-11 | 0.009 | 0.021 | 0.684 |
| rs810536 | A | G | -0.019 | 0.003 | 5.81E-12 | -0.004 | 0.022 | 0.839 |
| rs876039 | C | G | -0.028 | 0.003 | 7.96E-25 | 0.013 | 0.02 | 0.518 |
| rs8904 | A | G | 0.02 | 0.003 | 5.61E-14 | 0.014 | 0.019 | 0.444 |
| rs9390661 | C | G | 0.024 | 0.003 | 3.57E-13 | -0.008 | 0.023 | 0.714 |
| rs9554186 | A | T | 0.016 | 0.003 | 1.71E-09 | -0.02 | 0.019 | 0.291 |
| rs9787246 | A | G | 0.015 | 0.003 | 1.80E-08 | -0.009 | 0.019 | 0.628 |
| rs9896202 | C | T | 0.014 | 0.003 | 2.28E-08 | 0.007 | 0.018 | 0.708 |
| rs9908076 | T | C | 0.034 | 0.006 | 1.26E-08 | -0.038 | 0.039 | 0.324 |
| rs9931440 | C | A | 0.017 | 0.003 | 6.23E-11 | -0.015 | 0.019 | 0.417 |
| rs99780 | T | C | 0.036 | 0.003 | 1.78E-42 | -0.07 | 0.018 | 0 |

Supplementary Table 1. A shows the information of SNP between ALB and CRC. B shows the information of SNP between TP and CRC.

Supplementary Table 2

A

| SNP | beta.exposure | eaf.exposure | R^2 | F value |
| --- | --- | --- | --- | --- |
| rs10004084 | 0.030092 | 0.16526 | 0.000249834 | 78.78376418 |
| rs10042492 | -0.014739 | 0.43504 | 0.000106786 | 33.66948234 |
| rs10143115 | -0.014661 | 0.54022 | 0.000106777 | 33.66676825 |
| rs10213692 | 0.019841 | 0.24052 | 0.000143822 | 45.34863809 |
| rs10236582 | -0.022167 | 0.27836 | 0.000197411 | 62.24925289 |
| rs1030098 | 0.020357 | 0.38081 | 0.000195429 | 61.62427826 |
| rs10419198 | -0.073908 | 0.24966 | 0.002046539 | 646.527212 |
| rs10431419 | -0.016538 | 0.22283 | 9.47296E-05 | 29.86784838 |
| rs1043312 | 0.014211 | 0.34656 | 9.14668E-05 | 28.83900601 |
| rs10456852 | 0.026322 | 0.12956 | 0.000156271 | 49.27453723 |
| rs1079290 | 0.015474 | 0.42345 | 0.000116916 | 36.863979 |
| rs10793127 | 0.024629 | 0.092263 | 0.000101604 | 32.03556795 |
| rs10863570 | 0.022492 | 0.25511 | 0.000192267 | 60.6270473 |
| rs10919543 | -0.019189 | 0.31503 | 0.000158913 | 50.10768082 |
| rs11012732 | -0.023341 | 0.3307 | 0.00024117 | 76.0511562 |
| rs11057273 | -0.028641 | 0.90732 | 0.00013796 | 43.50006595 |
| rs11074901 | 0.01473 | 0.31238 | 9.3211E-05 | 29.38900139 |
| rs11078597 | 0.065424 | 0.18641 | 0.001298312 | 409.8456624 |
| rs11088253 | 0.013768 | 0.46312 | 9.42633E-05 | 29.72080369 |
| rs1110659 | 0.02205 | 0.23868 | 0.000176698 | 55.71656497 |
| rs111443054 | -0.028485 | 0.18411 | 0.000243765 | 76.86956913 |
| rs11217135 | -0.024493 | 0.44613 | 0.000296472 | 93.49516303 |
| rs11264233 | 0.019323 | 0.48849 | 0.00018659 | 58.83653507 |
| rs113177823 | 0.0428 | 0.054374 | 0.000188377 | 59.40010007 |
| rs114949263 | 0.056977 | 0.11154 | 0.000643425 | 202.9805081 |
| rs11589479 | 0.045527 | 0.1646 | 0.000570023 | 179.8113615 |
| rs11609805 | -0.02541 | 0.24326 | 0.000237715 | 74.96129938 |
| rs11656541 | 0.026835 | 0.65356 | 0.000326097 | 102.8408058 |
| rs117127664 | 0.037526 | 0.041048 | 0.000110862 | 34.95495128 |
| rs11736842 | 0.015911 | 0.63696 | 0.000117082 | 36.91641961 |
| rs117820542 | -0.041143 | 0.028246 | 9.29256E-05 | 29.29899302 |
| rs11895352 | -0.013656 | 0.4782 | 9.30659E-05 | 29.34325008 |
| rs11928797 | 0.027873 | 0.11709 | 0.000160633 | 50.65013078 |
| rs12215904 | -0.01996 | 0.18062 | 0.000117924 | 37.18181794 |
| rs1229492 | -0.017075 | 0.73291 | 0.000114146 | 35.99037801 |
| rs1229984 | 0.059701 | 0.977687 | 0.000155507 | 49.03381575 |
| rs12377600 | -0.018159 | 0.3464 | 0.000149315 | 47.08100968 |
| rs12563096 | 0.01427 | 0.66478 | 9.07582E-05 | 28.61556748 |
| rs1260326 | -0.059001 | 0.60704 | 0.001660789 | 524.4612462 |
| rs12710562 | 0.026315 | 0.18423 | 0.000208144 | 65.63452832 |
| rs12815728 | 0.018075 | 0.54087 | 0.000162261 | 51.16379893 |
| rs12881869 | -0.04948 | 0.071195 | 0.00032379 | 102.1130377 |
| rs13107325 | -0.055886 | 0.074885 | 0.00043274 | 136.4871355 |
| rs13108218 | -0.044248 | 0.61819 | 0.000924244 | 291.6522137 |
| rs13111128 | -0.018612 | 0.30062 | 0.000145662 | 45.92906074 |
| rs13389219 | -0.029364 | 0.39364 | 0.000411614 | 129.8213524 |
| rs138833981 | -0.085809 | 0.016171 | 0.000234289 | 73.88071221 |
| rs139278099 | 0.062656 | 0.041636 | 0.000313296 | 98.80252401 |
| rs139974673 | 0.15127 | 0.025096 | 0.001119701 | 353.3992767 |
| rs147651823 | -0.034933 | 0.046717 | 0.000108692 | 34.27069418 |
| rs149092986 | -0.054896 | 0.023997 | 0.000141163 | 44.51003627 |
| rs1497406 | 0.025056 | 0.58023 | 0.000305819 | 96.44395816 |
| rs1500187 | -0.023694 | 0.45557 | 0.000278486 | 87.82173811 |
| rs150783681 | -0.11931 | 0.020253 | 0.00056492 | 178.200755 |
| rs1532085 | 0.014174 | 0.6132 | 9.53023E-05 | 30.04844428 |
| rs157936 | -0.019043 | 0.30488 | 0.000153706 | 48.46557233 |
| rs1593357 | -0.020357 | 0.24512 | 0.000153361 | 48.3568454 |
| rs16950612 | -0.025444 | 0.10959 | 0.000126346 | 39.83765229 |
| rs17023530 | -0.038379 | 0.048199 | 0.000135145 | 42.6125303 |
| rs17580 | 0.094752 | 0.048858 | 0.000834426 | 263.28583 |
| rs1782455 | 0.019719 | 0.8385 | 0.000105311 | 33.2045989 |
| rs1791936 | 0.021346 | 0.60754 | 0.000217287 | 68.51801859 |
| rs1801282 | -0.050658 | 0.12024 | 0.000542924 | 171.2585427 |
| rs1880241 | 0.015573 | 0.49335 | 0.000121238 | 38.22676397 |
| rs1886839 | 0.014096 | 0.5982 | 9.55164E-05 | 30.11596195 |
| rs198426 | -0.016913 | 0.33512 | 0.000127472 | 40.1927212 |
| rs1986133 | 0.016108 | 0.68955 | 0.000111089 | 35.02644404 |
| rs1998528 | 0.015217 | 0.34623 | 0.000104828 | 33.05220409 |
| rs2060658 | 0.016588 | 0.55352 | 0.000136005 | 42.88343671 |
| rs2072442 | 0.014529 | 0.57233 | 0.000103337 | 32.58207647 |
| rs2115868 | -0.024382 | 0.16168 | 0.000161152 | 50.81382726 |
| rs2169387 | 0.057547 | 0.8992 | 0.000600333 | 189.3782673 |
| rs2200061 | -0.016504 | 0.213 | 9.13193E-05 | 28.79251218 |
| rs2227827 | -0.051242 | 0.047899 | 0.000239492 | 75.52187558 |
| rs2267867 | -0.016091 | 0.242 | 9.49906E-05 | 29.9501519 |
| rs2303695 | -0.025668 | 0.34793 | 0.000298951 | 94.27730139 |
| rs2304130 | 0.02707 | 0.085771 | 0.000114922 | 36.23506322 |
| rs234043 | 0.016912 | 0.71765 | 0.00011591 | 36.54667704 |
| rs2638315 | 0.035351 | 0.18211 | 0.000372273 | 117.4088782 |
| rs2702571 | 0.016766 | 0.64683 | 0.000128429 | 40.49447708 |
| rs2710804 | -0.016474 | 0.37709 | 0.000127497 | 40.20045316 |
| rs2785172 | -0.019156 | 0.60769 | 0.000174965 | 55.17016085 |
| rs2820446 | -0.023986 | 0.29913 | 0.000241237 | 76.07203131 |
| rs28601761 | -0.019962 | 0.41921 | 0.000194039 | 61.18574847 |
| rs28687959 | 0.016421 | 0.56183 | 0.000132763 | 41.86118919 |
| rs28688002 | 0.027203 | 0.22492 | 0.000258011 | 81.36308515 |
| rs2869876 | 0.017918 | 0.18453 | 9.66238E-05 | 30.46513678 |
| rs28929474 | 0.26514 | 0.020227 | 0.002786361 | 880.8995097 |
| rs2972145 | 0.021428 | 0.64679 | 0.000209792 | 66.15426007 |
| rs3099371 | 0.015872 | 0.59351 | 0.000121555 | 38.32667485 |
| rs3184504 | 0.021388 | 0.51817 | 0.000228421 | 72.02989783 |
| rs34562254 | -0.037537 | 0.097747 | 0.000248531 | 78.37291027 |
| rs34931250 | 0.03341 | 0.061215 | 0.000128294 | 40.45198152 |
| rs35123414 | 0.021697 | 0.13275 | 0.000108395 | 34.176881 |
| rs35676551 | -0.037895 | 0.058465 | 0.000158098 | 49.85078992 |
| rs36090025 | 0.017388 | 0.29934 | 0.000126824 | 39.98835665 |
| rs3740688 | 0.024193 | 0.54426 | 0.000290357 | 91.56642688 |
| rs3768321 | -0.028418 | 0.19703 | 0.000255534 | 80.58177331 |
| rs378740 | 0.01565 | 0.27137 | 9.68562E-05 | 30.53843418 |
| rs3810484 | 0.014777 | 0.44369 | 0.000107795 | 33.98779605 |
| rs390801 | 0.021268 | 0.26332 | 0.000175487 | 55.33493209 |
| rs4327724 | 0.031296 | 0.078888 | 0.000142341 | 44.88177321 |
| rs4410790 | 0.015033 | 0.63416 | 0.00010486 | 33.06237317 |
| rs4458838 | -0.015661 | 0.36968 | 0.000114303 | 36.03983535 |
| rs4499445 | -0.021739 | 0.65478 | 0.000213649 | 67.37059882 |
| rs45439091 | 0.031713 | 0.072593 | 0.000135416 | 42.69782136 |
| rs45512696 | 0.080056 | 0.17598 | 0.001858741 | 587.0890279 |
| rs459193 | 0.022894 | 0.74713 | 0.000198046 | 62.4496476 |
| rs4711399 | 0.021968 | 0.77921 | 0.000166052 | 52.35934567 |
| rs473919 | -0.018882 | 0.19619 | 0.000112449 | 35.45538696 |
| rs4782568 | -0.018428 | 0.45247 | 0.000168261 | 53.05597867 |
| rs4790875 | -0.020709 | 0.59435 | 0.000206796 | 65.2092125 |
| rs4804413 | 0.017594 | 0.42891 | 0.000151646 | 47.81596213 |
| rs4805129 | -0.021111 | 0.62941 | 0.00020791 | 65.56051886 |
| rs4805881 | -0.02704 | 0.66529 | 0.000325629 | 102.693187 |
| rs4833945 | 0.021705 | 0.11578 | 9.64592E-05 | 30.41322574 |
| rs4946811 | 0.015897 | 0.35713 | 0.000116041 | 36.58789068 |
| rs4970834 | -0.025389 | 0.18764 | 0.000196515 | 61.96659493 |
| rs55696240 | 0.017035 | 0.37843 | 0.000136518 | 43.04535601 |
| rs55722786 | 0.028148 | 0.28629 | 0.000323782 | 102.1105974 |
| rs55724869 | 0.014174 | 0.41358 | 9.74503E-05 | 30.72575872 |
| rs55846720 | -0.014796 | 0.56628 | 0.000107537 | 33.9065151 |
| rs56094005 | -0.046542 | 0.043798 | 0.000181436 | 57.21105373 |
| rs56188865 | 0.016247 | 0.37036 | 0.00012311 | 38.81712339 |
| rs58558667 | 0.023041 | 0.37416 | 0.00024863 | 78.40404085 |
| rs58579887 | 0.018348 | 0.40407 | 0.000162128 | 51.12188618 |
| rs59431480 | 0.085838 | 0.0095522 | 0.00013942 | 43.96042319 |
| rs59916403 | -0.024299 | 0.35213 | 0.0002694 | 84.95556762 |
| rs6031847 | -0.021265 | 0.26771 | 0.0001773 | 55.9065395 |
| rs60644673 | -0.017542 | 0.19469 | 9.64928E-05 | 30.42383738 |
| rs61552236 | -0.0161 | 0.27125 | 0.000102478 | 32.31109728 |
| rs61817641 | -0.018206 | 0.26915 | 0.000130401 | 41.11643771 |
| rs61983272 | -0.032856 | 0.32461 | 0.000473343 | 149.2996025 |
| rs62053895 | -0.013849 | 0.41445 | 9.309E-05 | 29.35083915 |
| rs631695 | 0.015489 | 0.58346 | 0.000116612 | 36.76819745 |
| rs6490409 | -0.019425 | 0.14363 | 9.28237E-05 | 29.26685846 |
| rs6549406 | -0.018975 | 0.73128 | 0.000141507 | 44.61859063 |
| rs6567095 | 0.01941 | 0.55434 | 0.000186149 | 58.6974085 |
| rs667172 | 0.018923 | 0.28615 | 0.000146289 | 46.12659132 |
| rs6693993 | 0.023539 | 0.53469 | 0.000275709 | 86.94554883 |
| rs6734238 | -0.016003 | 0.40207 | 0.000123136 | 38.82535515 |
| rs673751 | -0.015293 | 0.67774 | 0.000102161 | 32.21116263 |
| rs67694436 | 0.01512 | 0.35645 | 0.000104885 | 33.07022744 |
| rs6793835 | 0.016418 | 0.26438 | 0.000104846 | 33.05790277 |
| rs6794370 | -0.019261 | 0.80694 | 0.00011559 | 36.44588862 |
| rs6860245 | -0.021427 | 0.2484 | 0.000171432 | 54.05584898 |
| rs6871748 | 0.015224 | 0.27615 | 9.26576E-05 | 29.21450631 |
| rs6897617 | 0.018249 | 0.28838 | 0.000136685 | 43.09808002 |
| rs6912315 | 0.031344 | 0.052932 | 9.85005E-05 | 31.05690806 |
| rs6970593 | -0.025504 | 0.48766 | 0.000325029 | 102.5038815 |
| rs7031621 | -0.014376 | 0.5038 | 0.000103329 | 32.57939843 |
| rs72631343 | 0.021597 | 0.12744 | 0.000103733 | 32.70695435 |
| rs72789541 | 0.018236 | 0.29506 | 0.000138341 | 43.62032295 |
| rs72793380 | 0.02248 | 0.10516 | 9.51083E-05 | 29.98727619 |
| rs72818989 | 0.015766 | 0.54207 | 0.000123404 | 38.90973224 |
| rs72959041 | 0.051281 | 0.050215 | 0.000250843 | 79.10206664 |
| rs73038384 | -0.052059 | 0.031599 | 0.000165863 | 52.2997223 |
| rs73234873 | -0.014986 | 0.29494 | 9.34031E-05 | 29.44956659 |
| rs7366884 | 0.018621 | 0.27246 | 0.000137466 | 43.34434708 |
| rs7402977 | -0.016271 | 0.26675 | 0.000103565 | 32.65405368 |
| rs74538877 | 0.031123 | 0.056099 | 0.000102583 | 32.34418088 |
| rs74780677 | 0.093012 | 0.01564 | 0.000266378 | 84.00236366 |
| rs7591567 | 0.015616 | 0.29485 | 0.000101403 | 31.97226592 |
| rs76895963 | -0.071224 | 0.021056 | 0.00020913 | 65.94538489 |
| rs7731045 | -0.014222 | 0.38995 | 9.62334E-05 | 30.34202995 |
| rs77542162 | -0.19345 | 0.022953 | 0.001678504 | 530.0649527 |
| rs77849807 | 0.11375 | 0.015709 | 0.000400133 | 126.1989691 |
| rs78444263 | -0.03521 | 0.071231 | 0.000164036 | 51.72341377 |
| rs78961851 | -0.024799 | 0.10258 | 0.000113229 | 35.70122233 |
| rs79295634 | 0.03133 | 0.066607 | 0.000122049 | 38.48268302 |
| rs800545 | -0.016169 | 0.74677 | 9.88777E-05 | 31.17585811 |
| rs8041057 | -0.023221 | 0.71393 | 0.000220252 | 69.4532514 |
| rs8072215 | -0.033891 | 0.23131 | 0.000408455 | 128.8246482 |
| rs854796 | 0.015472 | 0.68333 | 0.0001036 | 32.66498408 |
| rs872926 | -0.035827 | 0.20696 | 0.00042134 | 132.8900769 |
| rs879620 | -0.014193 | 0.6154 | 9.53554E-05 | 30.06517416 |
| rs900400 | 0.022534 | 0.39764 | 0.00024325 | 76.70709874 |
| rs9265945 | -0.023445 | 0.42942 | 0.000269358 | 84.94218239 |
| rs930340 | -0.018877 | 0.81033 | 0.000109536 | 34.5367303 |
| rs9391997 | -0.016206 | 0.53012 | 0.000130841 | 41.25501771 |
| rs9638180 | -0.024469 | 0.19723 | 0.000189595 | 59.78414615 |
| rs9917677 | -0.015506 | 0.25289 | 9.08544E-05 | 28.64589243 |
| rs992367 | 0.015386 | 0.59857 | 0.000113764 | 35.87011511 |
| rs9976946 | 0.038971 | 0.962177 | 0.000110541 | 34.85372388 |

B.

| SNP | beta.exposure | eaf.exposure | R^2 | F value |
| --- | --- | --- | --- | --- |
| rs10004084 | 0.035473 | 0.16525 | 0.000347155 | 109.3637483 |
| rs10065637 | -0.039914 | 0.20469 | 0.000518697 | 163.4322501 |
| rs10069690 | -0.021817 | 0.25761 | 0.00018206 | 57.34463971 |
| rs10111287 | 0.018742 | 0.2827 | 0.000142459 | 44.86929431 |
| rs10160596 | -0.020316 | 0.2665 | 0.000161363 | 50.82444957 |
| rs1020048 | -0.02673 | 0.8099 | 0.000220009 | 69.30041768 |
| rs10419198 | -0.074871 | 0.24964 | 0.002100105 | 662.7549785 |
| rs10444863 | -0.028355 | 0.2072 | 0.000264145 | 83.20631471 |
| rs1047891 | -0.025697 | 0.31562 | 0.00028527 | 89.86269569 |
| rs10745495 | -0.017412 | 0.22333 | 0.000105175 | 33.1249668 |
| rs10750400 | 0.018489 | 0.5244 | 0.000170515 | 53.70742035 |
| rs10846690 | 0.026178 | 0.84581 | 0.000178744 | 56.30000645 |
| rs10882100 | 0.015152 | 0.52093 | 0.00011459 | 36.09083217 |
| rs10887917 | 0.018342 | 0.37425 | 0.000157575 | 49.63103637 |
| rs10898811 | -0.017802 | 0.44734 | 0.000156698 | 49.35490011 |
| rs11099882 | -0.020536 | 0.34825 | 0.000191441 | 60.29980187 |
| rs11102009 | -0.019592 | 0.53071 | 0.000191199 | 60.22378116 |
| rs11118320 | 0.017259 | 0.56332 | 0.000146548 | 46.15749579 |
| rs11128592 | 0.026298 | 0.13313 | 0.000159627 | 50.27748411 |
| rs11138299 | -0.028091 | 0.084542 | 0.000122145 | 38.47044862 |
| rs11217863 | 0.023259 | 0.1161 | 0.000111032 | 34.96990992 |
| rs112733823 | 0.033472 | 0.11698 | 0.00023146 | 72.90794809 |
| rs113810201 | 0.025186 | 0.1141 | 0.000128239 | 40.38994859 |
| rs114310991 | -0.11455 | 0.014741 | 0.000381151 | 120.0775911 |
| rs11447452 | 0.024485 | 0.80663 | 0.000187022 | 58.9079316 |
| rs114949263 | 0.030307 | 0.11156 | 0.000182076 | 57.34960726 |
| rs11594976 | -0.014109 | 0.49255 | 9.95098E-05 | 31.34065908 |
| rs11620783 | 0.017963 | 0.43125 | 0.000158284 | 49.85467212 |
| rs11711621 | 0.018268 | 0.24485 | 0.000123409 | 38.86850165 |
| rs118083884 | -0.1233 | 0.01656 | 0.000495181 | 156.0192987 |
| rs11879090 | -0.014306 | 0.4492 | 0.000101275 | 31.89649519 |
| rs1196837 | -0.01698 | 0.52656 | 0.000143753 | 45.27719146 |
| rs12142550 | 0.018383 | 0.25135 | 0.00012718 | 40.05664666 |
| rs12363520 | -0.017376 | 0.2296 | 0.000106811 | 33.64054501 |
| rs12377600 | -0.016946 | 0.3464 | 0.000130033 | 40.95525806 |
| rs12386224 | 0.015394 | 0.28564 | 9.67095E-05 | 30.45860293 |
| rs12434109 | -0.016408 | 0.50317 | 0.000134606 | 42.39563731 |
| rs12453969 | 0.022134 | 0.31689 | 0.000212104 | 66.80976951 |
| rs12505932 | 0.014586 | 0.43536 | 0.000104598 | 32.94328261 |
| rs12544863 | -0.020686 | 0.39394 | 0.000204328 | 64.3600466 |
| rs1260326 | -0.042401 | 0.60701 | 0.000857748 | 270.3529284 |
| rs1271309 | 0.018793 | 0.83688 | 9.64257E-05 | 30.36920113 |
| rs12722497 | 0.036789 | 0.088111 | 0.000217489 | 68.50643962 |
| rs12881869 | -0.032239 | 0.071167 | 0.000137407 | 43.27807735 |
| rs13005282 | -0.028384 | 0.36128 | 0.000371819 | 117.1364344 |
| rs13020178 | 0.014268 | 0.49841 | 0.000101787 | 32.05788638 |
| rs13108218 | -0.034971 | 0.61817 | 0.00057733 | 181.9171894 |
| rs13266875 | -0.028025 | 0.098813 | 0.000139878 | 44.05648633 |
| rs13278421 | -0.019344 | 0.14889 | 9.48361E-05 | 29.86852926 |
| rs13322435 | 0.021883 | 0.40102 | 0.00023005 | 72.46375836 |
| rs13380871 | 0.057364 | 0.034678 | 0.00022031 | 69.39523488 |
| rs13425999 | -0.034888 | 0.070988 | 0.000160542 | 50.56581357 |
| rs138833981 | -0.073713 | 0.016173 | 0.000172913 | 54.4629872 |
| rs139974673 | 0.10378 | 0.025107 | 0.000527241 | 166.1257692 |
| rs140118846 | -0.074025 | 0.020221 | 0.000217129 | 68.3928585 |
| rs1448187 | -0.040285 | 0.70016 | 0.000681402 | 214.7328512 |
| rs1458019 | 0.028532 | 0.26694 | 0.000318601 | 100.3655936 |
| rs148393876 | -0.052179 | 0.062475 | 0.000318941 | 100.472688 |
| rs149914551 | -0.045434 | 0.035264 | 0.000140453 | 44.23763153 |
| rs1561721 | -0.017989 | 0.74103 | 0.000124202 | 39.11849103 |
| rs1571878 | -0.017135 | 0.563 | 0.000144473 | 45.50400865 |
| rs157936 | -0.020555 | 0.30486 | 0.000179076 | 56.40457422 |
| rs1611236 | -0.038357 | 0.3225 | 0.000642922 | 202.5986048 |
| rs1657792 | 0.019326 | 0.57158 | 0.00018292 | 57.61545807 |
| rs16826069 | -0.022624 | 0.21332 | 0.00017179 | 54.10930325 |
| rs1689789 | -0.019351 | 0.35986 | 0.000172522 | 54.33994047 |
| rs17090693 | -0.018407 | 0.25223 | 0.000127809 | 40.25456813 |
| rs17711850 | 0.02817 | 0.36462 | 0.000367687 | 115.8340641 |
| rs17764730 | -0.022438 | 0.24788 | 0.000187727 | 59.12992227 |
| rs1801282 | -0.040815 | 0.12022 | 0.000352387 | 111.0126159 |
| rs181242111 | 0.035061 | 0.11837 | 0.00025657 | 80.81963892 |
| rs1975161 | -0.018838 | 0.63249 | 0.000164977 | 51.96284526 |
| rs2014842 | 0.018712 | 0.18192 | 0.000104219 | 32.82394406 |
| rs201822981 | -0.021135 | 0.66113 | 0.000200149 | 63.04349527 |
| rs2068888 | -0.016483 | 0.44867 | 0.000134413 | 42.33488768 |
| rs2236295 | -0.016772 | 0.40483 | 0.000135554 | 42.69442431 |
| rs2273215 | -0.01413 | 0.45907 | 9.91595E-05 | 31.23030535 |
| rs2283620 | -0.015569 | 0.33999 | 0.000108785 | 34.26211773 |
| rs2293445 | -0.016304 | 0.39034 | 0.000126517 | 39.84766718 |
| rs2371108 | 0.014331 | 0.38628 | 9.73768E-05 | 30.66879007 |
| rs2396746 | 0.016651 | 0.53699 | 0.000137869 | 43.42361246 |
| rs2412974 | -0.02717 | 0.35916 | 0.000339818 | 107.0516381 |
| rs2442517 | -0.014114 | 0.46835 | 9.92034E-05 | 31.2441356 |
| rs2466574 | 0.01769 | 0.29301 | 0.000129653 | 40.83537514 |
| rs2516655 | -0.033143 | 0.6528 | 0.000497936 | 156.8876147 |
| rs2518710 | -0.026381 | 0.079155 | 0.000101456 | 31.95363858 |
| rs2532387 | -0.034635 | 0.21862 | 0.000409838 | 129.1187911 |
| rs2549508 | 0.0249 | 0.87074 | 0.000139567 | 43.95829284 |
| rs2681416 | 0.015197 | 0.32851 | 0.000101891 | 32.09053686 |
| rs2720660 | -0.016684 | 0.61979 | 0.000131189 | 41.31942795 |
| rs2774947 | 0.036002 | 0.911693 | 0.000208702 | 65.738004 |
| rs28368955 | -0.023531 | 0.16556 | 0.000152989 | 48.18663581 |
| rs28499538 | -0.016027 | 0.28147 | 0.000103899 | 32.72317817 |
| rs28505206 | 0.030383 | 0.13065 | 0.000209699 | 66.05190074 |
| rs28929474 | 0.1393 | 0.020227 | 0.000769111 | 242.3941773 |
| rs2972156 | 0.018834 | 0.64636 | 0.000162163 | 51.07639981 |
| rs303929 | -0.014743 | 0.32192 | 9.48922E-05 | 29.88619994 |
| rs3087243 | -0.019972 | 0.45025 | 0.000197466 | 62.19804017 |
| rs34215892 | -0.043113 | 0.028482 | 0.000102865 | 32.39748946 |
| rs34322 | -0.016939 | 0.52293 | 0.000143163 | 45.09124636 |
| rs34562254 | 0.11755 | 0.097756 | 0.002437489 | 769.4872567 |
| rs34592828 | -0.05516 | 0.045516 | 0.000264369 | 83.27697576 |
| rs34642860 | 0.015184 | 0.31096 | 9.87987E-05 | 31.11667051 |
| rs34936565 | -0.024381 | 0.20226 | 0.000191825 | 60.42081463 |
| rs35049983 | -0.017064 | 0.32527 | 0.00012781 | 40.25502355 |
| rs350822 | 0.022852 | 0.74613 | 0.000197836 | 62.31449829 |
| rs352139 | 0.014508 | 0.53936 | 0.000104589 | 32.94046766 |
| rs35986445 | -0.021146 | 0.18726 | 0.000136108 | 42.86877601 |
| rs35996865 | -0.020494 | 0.20154 | 0.000135175 | 42.57507794 |
| rs374039502 | 0.10686 | 0.020627 | 0.000461365 | 145.3596301 |
| rs3740688 | 0.017562 | 0.5443 | 0.000153001 | 48.19040993 |
| rs3755172 | 0.024276 | 0.84612 | 0.000153461 | 48.33524317 |
| rs3756772 | 0.022509 | 0.40081 | 0.000243358 | 76.65669106 |
| rs3781351 | 0.01397 | 0.44904 | 9.65668E-05 | 30.41366173 |
| rs3790315 | -0.015839 | 0.58124 | 0.000122125 | 38.46432397 |
| rs3798556 | -0.018203 | 0.18 | 9.78143E-05 | 30.80659061 |
| rs3803286 | -0.047917 | 0.66679 | 0.001020273 | 321.6314648 |
| rs3803800 | -0.042923 | 0.7885 | 0.0006145 | 193.6368556 |
| rs3810504 | 0.026302 | 0.12143 | 0.000147608 | 46.49142747 |
| rs3811647 | 0.019458 | 0.34011 | 0.000169949 | 53.52910966 |
| rs3811671 | -0.017769 | 0.20624 | 0.000103376 | 32.55830907 |
| rs3812207 | -0.014808 | 0.37781 | 0.000103091 | 32.46855197 |
| rs38246 | -0.017769 | 0.7333 | 0.000123498 | 38.8967372 |
| rs3849768 | 0.020086 | 0.21062 | 0.000134154 | 42.25321596 |
| rs3870401 | -0.016302 | 0.27149 | 0.000105124 | 33.10896838 |
| rs3891176 | -0.074725 | 0.24138 | 0.002044972 | 645.3202707 |
| rs41444548 | 0.045593 | 0.075969 | 0.000291843 | 91.93373697 |
| rs42032 | -0.016396 | 0.26211 | 0.000103987 | 32.75103713 |
| rs4482257 | -0.015858 | 0.26934 | 9.8979E-05 | 31.17344958 |
| rs4649017 | 0.014257 | 0.38404 | 9.61646E-05 | 30.28697476 |
| rs4729594 | -0.014902 | 0.5983 | 0.000106743 | 33.61902952 |
| rs4790877 | -0.032871 | 0.58876 | 0.000523226 | 164.8601281 |
| rs4805881 | -0.016043 | 0.66526 | 0.00011463 | 36.10346092 |
| rs4845107 | -0.020668 | 0.85104 | 0.000108305 | 34.11084204 |
| rs4852257 | 0.014667 | 0.5759 | 0.000105082 | 33.09576569 |
| rs4938496 | -0.029174 | 0.30003 | 0.000357492 | 112.6212154 |
| rs4940573 | 0.03994 | 0.13193 | 0.00036538 | 115.1070707 |
| rs5001409 | -0.022233 | 0.38948 | 0.000235078 | 74.04779952 |
| rs55772024 | -0.01786 | 0.24231 | 0.000117127 | 36.88973471 |
| rs55844407 | -0.01946 | 0.19302 | 0.000117972 | 37.15616234 |
| rs55865752 | 0.016503 | 0.24303 | 0.000100206 | 31.55998112 |
| rs56031211 | -0.018215 | 0.18261 | 9.90472E-05 | 31.19493643 |
| rs567743 | 0.016982 | 0.70806 | 0.000119226 | 37.55104083 |
| rs58432776 | 0.02075 | 0.38034 | 0.000202951 | 63.9261686 |
| rs58895965 | 0.048553 | 0.17633 | 0.000684765 | 215.7932945 |
| rs59418918 | 0.014347 | 0.41214 | 9.97403E-05 | 31.41326311 |
| rs59441438 | -0.023695 | 0.12751 | 0.000124925 | 39.34605971 |
| rs61237993 | -0.021743 | 0.12696 | 0.000104802 | 33.00763167 |
| rs61830291 | 0.030004 | 0.097205 | 0.000158003 | 49.76609314 |
| rs62045817 | -0.025025 | 0.1525 | 0.000161878 | 50.98669841 |
| rs62131205 | -0.039589 | 0.078797 | 0.000227533 | 71.67072162 |
| rs62441844 | 0.017692 | 0.20718 | 0.000102827 | 32.38544032 |
| rs631864 | -0.025716 | 0.47072 | 0.000329522 | 103.8070768 |
| rs6447324 | -0.022344 | 0.18447 | 0.000150216 | 47.31312536 |
| rs6557616 | 0.021893 | 0.78901 | 0.000159582 | 50.26354374 |
| rs6600250 | -0.034261 | 0.49943 | 0.000586907 | 184.9368001 |
| rs66477686 | -0.025176 | 0.27601 | 0.000253315 | 79.79390716 |
| rs6665912 | 0.029204 | 0.22394 | 0.000296443 | 93.38331246 |
| rs6720027 | 0.015758 | 0.40379 | 0.00011956 | 37.65631294 |
| rs679574 | 0.037538 | 0.51071 | 0.000704227 | 221.9308981 |
| rs6833591 | -0.018088 | 0.34776 | 0.000148422 | 46.74783238 |
| rs6903640 | -0.021443 | 0.63804 | 0.000212378 | 66.89608174 |
| rs6924387 | 0.025174 | 0.40943 | 0.000306468 | 96.54226331 |
| rs6942338 | 0.046896 | 0.916311 | 0.000337297 | 106.2571716 |
| rs7005025 | -0.017822 | 0.63327 | 0.000147529 | 46.466629 |
| rs71393469 | -0.029173 | 0.10392 | 0.000158503 | 49.92359004 |
| rs7140 | -0.018445 | 0.69496 | 0.000144246 | 45.43237382 |
| rs71423603 | -0.021189 | 0.12478 | 9.80648E-05 | 30.88549649 |
| rs71610255 | -0.030383 | 0.12519 | 0.000202197 | 63.68855079 |
| rs7197422 | 0.016377 | 0.40874 | 0.000129636 | 40.83001136 |
| rs7228151 | 0.019739 | 0.20484 | 0.000126926 | 39.9763899 |
| rs72665951 | 0.016749 | 0.38123 | 0.00013235 | 41.68505951 |
| rs72704062 | -0.095481 | 0.025233 | 0.00044847 | 141.295163 |
| rs72799341 | 0.021491 | 0.24031 | 0.000168636 | 53.11578507 |
| rs72832890 | 0.025779 | 0.095066 | 0.000114342 | 36.01245925 |
| rs72835315 | 0.043623 | 0.058222 | 0.000208688 | 65.73341872 |
| rs7287486 | -0.061929 | 0.024818 | 0.00018564 | 58.47228697 |
| rs7295713 | -0.01415 | 0.58174 | 9.74357E-05 | 30.68734505 |
| rs7304603 | -0.014364 | 0.53929 | 0.000102525 | 32.29045666 |
| rs7421377 | 0.019993 | 0.37794 | 0.000187949 | 59.19997989 |
| rs74922662 | 0.03423 | 0.047866 | 0.000106799 | 33.63676746 |
| rs7502910 | 0.024976 | 0.50201 | 0.000311895 | 98.25238392 |
| rs750598 | -0.022396 | 0.33767 | 0.000224356 | 70.66984342 |
| rs75328154 | -0.018296 | 0.248 | 0.000124857 | 39.32465416 |
| rs75478023 | -0.035743 | 0.073107 | 0.000173141 | 54.53491335 |
| rs75899192 | -0.032422 | 0.056099 | 0.000111325 | 35.06213669 |
| rs76428106 | 0.12958 | 0.013121 | 0.000434847 | 137.0012553 |
| rs7659414 | -0.018333 | 0.42445 | 0.000164213 | 51.722184 |
| rs7676237 | 0.014578 | 0.33699 | 9.49649E-05 | 29.90908233 |
| rs76895963 | -0.077409 | 0.021058 | 0.000247051 | 77.82034697 |
| rs77542162 | -0.096717 | 0.022963 | 0.000419735 | 132.2380561 |
| rs7774393 | 0.020612 | 0.6835 | 0.000183816 | 57.89768481 |
| rs77849807 | 0.12582 | 0.015709 | 0.000489555 | 154.2456546 |
| rs7821812 | -0.036943 | 0.20732 | 0.000448573 | 141.3276497 |
| rs78484938 | -0.036913 | 0.037433 | 9.81916E-05 | 30.92543333 |
| rs7942247 | -0.017344 | 0.5044 | 0.000150396 | 47.36953108 |
| rs7966399 | 0.019943 | 0.27414 | 0.000158284 | 49.85444179 |
| rs7995886 | 0.01574 | 0.38756 | 0.000117609 | 37.04178363 |
| rs8092360 | -0.020732 | 0.20051 | 0.000137804 | 43.40304352 |
| rs810536 | -0.019018 | 0.70801 | 0.000149543 | 47.10108007 |
| rs876039 | -0.027838 | 0.31679 | 0.000335453 | 105.675986 |
| rs8904 | 0.019644 | 0.36087 | 0.000178004 | 56.06683056 |
| rs9390661 | 0.024036 | 0.18296 | 0.000172724 | 54.40361271 |
| rs9554186 | 0.016236 | 0.34163 | 0.000118581 | 37.34775324 |
| rs9787246 | 0.014545 | 0.39042 | 0.000100698 | 31.71486097 |
| rs9896202 | 0.014056 | 0.49936 | 9.87854E-05 | 31.11247478 |
| rs9908076 | 0.034196 | 0.046133 | 0.000102915 | 32.41333335 |
| rs9931440 | 0.016906 | 0.45033 | 0.000141496 | 44.56613364 |
| rs99780 | 0.035946 | 0.35134 | 0.000588947 | 185.5797416 |

Supplementary Table 2. A shows the F value of SNP between ALB and CRC. B shows the F value of SNP between TP and CRC.
